# dotears: Scalable and consistent directed acyclic graph estimation using observational and interventional data

**DOI:** 10.1016/j.isci.2024.111673

**Published:** 2024-12-24

**Authors:** Albert Xue, Jingyou Rao, Sriram Sankararaman, Harold Pimentel

**Affiliations:** 1 Bioinformatics Indepartmental Program, UCLA, Los Angeles, CA 90024, USA; 2 Department of Computer Science, UCLA, Los Angeles, CA 90024, USA; 3 Department of Computational Medicine, David Geffen School of Medicine, UCLA, Los Angeles, CA 90024, USA; 4 Department of Human Genetics, David Geffen School of Medicine, UCLA, Los Angeles, CA 90024, USA

**Keywords:** Gene network, Bioinformatics, Biocomputational method

## Abstract

New assays such as Perturb-seq link parallel CRISPR interventions to transcriptomic readouts, providing insight into gene regulatory networks. Causal regulatory networks can be represented by directed acyclic graphs (DAGs), but lack of identifiability and a combinatorial solution space complicate learning DAGs from observational data. Score-based methods have improved the practical scalability of inferring DAGs, but are sensitive to error variance structure. Furthermore, correction for error variance is difficult without prior knowledge of structure. We present dotears [doo-tairs], a continuous optimization framework leveraging observational and interventional data to infer causal structure, assuming a linear Structural Equation Model. dotears exploits structural consequences of hard interventions to estimate and correct for error variance structure. dotears is a provably consistent estimator of the true DAG under mild assumptions and outperforms other state-of-the-art methods in varied simulations. In real data, differential expression tests and high-confidence protein-protein interactions validate dotears-inferred edges with higher precision and recall than others.

## Highlights

• Continuous causal structure learning which incorporates interventional data
• Interventions enable robust identifiability of structure
• State-of-the-art performance in simulations and real data
• Estimator is statistically consistent under mild assumptions

## Graphical abstract

**Figure:**
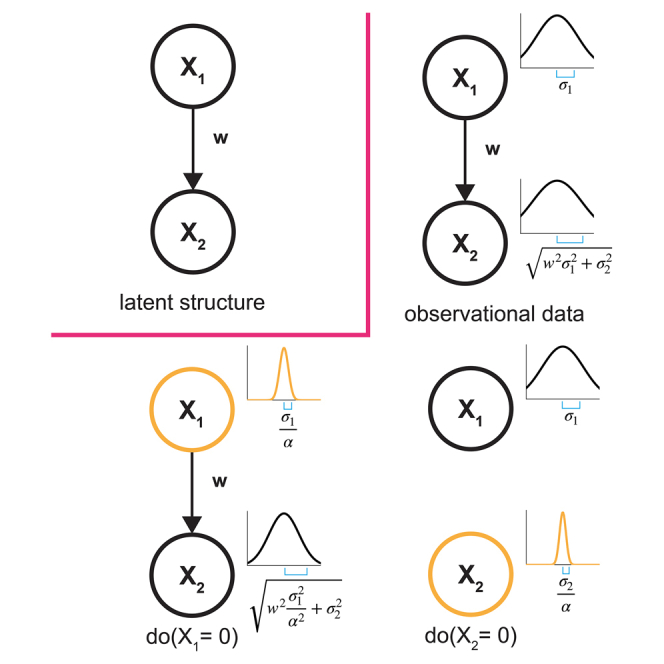

## Introduction

Understanding gene regulatory networks can identify mechanisms and pathways linking GWAS significant variants to phenotype. Recent efforts to map regulatory networks through *trans*-eQTLs are partly limited by power; for example, the GTEx project finds only 143 *trans*-eQTLs in 838 individuals.^1^ On the other hand, Võsa et al. detect almost 60,000 *trans*-eQTLs in ∼31,000 individuals, across more than a third of trait-associated variants.^2^ These results imply that *trans*-regulatory relationships are pervasive, but our ability to detect small eQTL effects is often limited by small sample size regimes, observational data, and a high multiple testing burden. Importantly, since rare tissues are unlikely to be sampled at sufficient sample sizes, capturing gene regulation events across a wide array of cell types and tissues requires another experimental method.

High-throughput genomic technologies such as Perturb-seq provide a natural alternative for learning gene regulatory networks. Perturb-seq links high-dimensional transcriptomic readouts to known, highly parallel CRISPR interventions, allows the direct interrogation of causal regulatory relationships, and has scaled genome-wide.^3^^,^^4^^,^^5^ In particular, the effects of CRISPR gene interventions are large in comparison to QTL effects, facilitating inference of downstream regulatory relationships. Notably, analogous experiments have already mapped gene-gene networks in yeast.^6^^,^^7^^,^^8^

The inference of gene regulatory networks can be treated as a causal structure learning problem, which considers learning relationships between variables in the form of a Directed Acyclic Graph (DAG). Here, directedness gives a natural causal interpretation, while acyclicity ensures that the causal interpretation is valid. For example, in the mediator DAG i→j→k, we understand that gene *j* has a direct causal regulatory effect on gene *k*, and similarly that gene *i* (indirectly) affects gene *k* only through its direct effect on gene *j*.

Identifiability and scalability are the primary difficulties in learning DAGs from data. For identifiability, distinct DAGs may contain the same conditional independence relationships in *observational* data, and DAGs are only identifiable up to Markov equivalence.^9^^,^^10^^,^^11^ For scalability, Zheng et al. introduced DAGs with NO TEARS, a method that allows for continuous optimization through a continuous, differentiable acyclicity constraint.^12^ As a result, DAGs with NO TEARS avoids combinatorial characterizations of DAGs, and is a fundamental methodological building block for many structure learning methods.^13^^,^^14^ However, NO TEARS and related methods infer DAGs whose topological order follows increasing marginal variance, and re-scaling data can change or reverse their inferences.^15^ This poses issues for inferring gene regulatory networks from data, where the scale of exogenous error between genes is likely not uniform.

Fundamentally, this is still an issue of identifiability. Because NO TEARS uses observational data, it must choose a single member of a class of Markov equivalent DAGs. The “tiebreaker” is then a function of the variance. However, we show that interventional data can correct for variance sensitivity in NO TEARS and related methods, and further that this correction is sufficient for the consistent estimation of structure.

Explicitly, exogenous error in the linear SEM drives variance sensitivity. Let *X* be a *p*-dimensional random vector (e.g., the distribution of gene expression across *p* genes), $W_{0}\in R^{p\times p}$ the weighted adjacency matrix of the true DAG, and ϵ a *p*-dimensional random vector specifying the exogenous error. The linear SEM gives the autoregressive formulation

$$X=XW_{0}+\epsilon \text{,}$$

where $\Omega_{0}:=\text{Cov}\left(\epsilon \right)=\text{diag}\left(\sigma_{1}^{2},\ldots ,\sigma_{p}^{2}\right)$. For any node *j* we then have

(Equation 1) $$\text{Var}\left(X_{j}\right)=\sum_{i=1}^{p}w_{ij}^{2}\text{Var}\left(X_{i}\right)+\sigma_{j}^{2}\text{.}$$

$\text{Var}\left(X_{i}\right)$ is itself a linear function of $\sigma_{1}^{2},\ldots ,\sigma_{p}^{2}$. Critically, parental error variances thus propagate to downstream nodes to provide a signal of structure. However, in observational data the exact relationship between $W_{0}$, $\Omega_{0}$, and Var(X) is unidentifiable.

Consequently, given $\Omega_{0}$ then $W_{0}$ is recoverable even in observational data.^16^ However, the estimation of $\Omega_{0}$ is difficult without *a priori* knowledge of $W_{0}$. Previous methods either ignore exogenous variance structure or use the conditional estimate ${\hat{\Omega }}_{0}|W$^12^^,^^13^. We show that both procedures are sensitive to exogenous variance structure even in the simplest two node DAG.

*Hard* interventions^17^^,^^18^ remove upstream variance in the linear SEM to allow the marginal estimation of $\Omega_{0}$. We show that the naive incorporation of interventional data into the NO TEARS framework, without the estimation of $\Omega_{0}$, is insufficient for structural recovery. Finally, we show correction by this marginal estimate is sufficient for structural recovery.

Accordingly, we present dotears, a novel optimization framework for structure learning. dotears uses **1.)** a novel marginal estimation procedure for $\Omega_{0}$ using the structural consequences of interventions, and **2.)** joint estimation of the causal DAG from observational and interventional data, given the estimated ${\hat{\Omega }}_{0}$. dotears provides a simple model that we show, by extending results from Loh and Buhlmann,^16^ is a provably consistent estimator of the true DAG under mild assumptions. In simulations, dotears corrects for exogenous variance structure and is robust to reasonable violations of its modeling assumptions. We also apply dotears to the Perturb-seq experiment in Replogle et al*.*^5^ dotears infers a sparse set of edges that validate with high precision in differential expression tests and in an orthogonal set of protein-protein interactions. In both simulations and real data, dotears outperforms all other tested methods in all used metrics.

### Model

We represent a gene regulatory network as G=([p],E), a DAG on *p* nodes with node set [p]:={1…p} and edge set E. We represent E with the weighted adjacency matrix $W\in D\subset R^{p\times p}$, where $D\subset R^{p\times p}$ is the set of weighted adjacency matrices on *p* nodes whose support is a DAG. We denote the parent set of node *i* in the observational setting as Pa(i). For $w_{ij}$ the i,j entry in *W*, $\left|w_{ij}\right|>0$ indicates an edge i→j with weight $w_{ij}$, equivalently denoted $i\overset{w_{ij}}{\to }j$, or equivalently an inferred gene regulatory event between genes *i* and *j*. k=0,1,…,p indexes the intervention, where k=0 is reserved for the observational system, and k≠0 denotes intervention on node *k*. Similarly, we denote ${\left(\cdot \right)}^{\left(0\right)}$ for observational quantities, and ${\left(\cdot \right)}^{\left(k\right)}$ for quantities under intervention on node *k*. For brevity, a variable without a superscript is assumed to be observational; for example, $X\equiv X^{\left(0\right)}$. X (bolded) denotes $n_{0}$ samples drawn from the *p*-dimensional random vector *X* (unbolded), and ϵ (bolded) denotes $n_{0}$ samples drawn from the *p*-dimensional random vector ϵ (unbolded). Similarly, if $X^{\left(k\right)}$ is a *p*-dimensional random vector, then $X^{\left(k\right)}\in R^{n_{k}\times p}$ represents $n_{k}$ observations of *X*. We denote the total sample size $n:=\sum_{k=0}^{p}n_{k}$, and the true weighted adjacency matrix $W_{0}$.

The linear SEM is an autoregressive representation of $X^{\left(k\right)}$ and weighted adjacency matrix $W_{0}^{\left(k\right)}$,

(Equation 2) $$X^{\left(k\right)}=X^{\left(k\right)}W_{0}^{\left(k\right)}+\epsilon^{\left(k\right)},k=0,\ldots ,p\text{.}$$

Here, $W_{0}^{\left(k\right)}$ is permutation-similar to a strictly upper triangular matrix, representing the constraint $W_{0}^{\left(k\right)}\in D$. For each *k*, $\epsilon^{\left(k\right)}$ is a *p*-dimensional random vector such that $E\epsilon^{\left(k\right)}=0_{p}$, and $\Omega_{0}^{\left(k\right)}:=\text{Cov}\left(\epsilon^{\left(k\right)}\right)$. Denote $\epsilon_{i}$ as the *i*th element of ϵ. Then $\epsilon_{i}^{\left(k\right)}$ is the exogenous error on node *i*, such that $E\epsilon_{i}^{\left(k\right)}=0$ and $\epsilon_{i}^{\left(k\right)}$$\epsilon_{j}^{\left(k\right)}$ for i≠j. We further define

$$\Omega_{0}:=\text{Cov}\left(\epsilon^{\left(0\right)}\right)=\text{diag}\left(\sigma_{1}^{2},\sigma_{2}^{2},\cdots ,\sigma_{p}^{2}\right)\text{.}$$

Motivated by recent work on a genome-wide screen that performs known single interventions on all protein-coding genes,^5^ we consider the linear SEM with known single interventions on all *p* nodes. Accordingly, we obtain a system of p+1 structural equations

$$\begin{array}{l} X^{\left(0\right)}=X^{\left(0\right)}W^{\left(0\right)}+\epsilon^{\left(0\right)}\\ \vdots \\ X^{\left(p\right)}=X^{\left(p\right)}W^{\left(p\right)}+\epsilon^{\left(p\right)}\text{.} \end{array}$$

In this setting we have complicated our problem. Before, with the single data matrix X, we inferred a single *W*; now, with the p+1 data matrices $X^{\left(0\right)},X^{\left(1\right)},\ldots ,X^{\left(p\right)}$, it seems that we must infer p+1 adjacency matrices $W^{\left(0\right)},W^{\left(1\right)},\ldots ,W^{\left(p\right)}$. Our model assumes hard interventions, i.e., that an intervention on node *k* removes causal influences from observational parents of *k*.^17^^,^^18^ Hard interventions relax the do operation $do\left(X_{k}^{\left(k\right)}=0\right)$ to allow for residual noise, modeling the limit of experimental interventional efficacy combined with a noisy readout.

**Assumption 1.** For an intervention k≠0, if in the observational setting

$$X_{k}^{\left(0\right)}=\sum_{i\in Pa\left(k\right)}W_{ik}X_{i}^{\left(0\right)}+\epsilon_{k}^{\left(0\right)}\text{,}$$

then upon intervention on k

$$X_{k}^{\left(k\right)}=\epsilon_{k}^{\left(k\right)}\text{.}$$

Under Assumption 1, we can relate $W^{\left(0\right)}$ to $W^{\left(k\right)}$ by setting the *k*th column of $W^{\left(0\right)}$ to ${\vec{0}}_{p}$, giving

(Equation 3) $$W_{ij}^{\left(k\right)}:=\left\{ \begin{array}{cc} W_{ij} & j\neq k\\ 0 & j=k \end{array}\right.\text{.}$$

This gives p+1 data matrices $X^{\left(k\right)}$ to jointly infer a single weighted adjacency matrix *W*. We now characterize the p+1 exogenous variance structures $\Omega_{0}^{\left(k\right)}$. We assume that the exogenous variance of non-targeted nodes j≠k is invariant, modeling a system where the effects of CRISPR interventions are restricted to the target.

**Assumption 2.** For an intervention k≠0, $Var\left(\epsilon_{j}^{\left(k\right)}\right)=Var\left(\epsilon_{j}^{\left(0\right)}\right)=\sigma_{j}^{2}$ for j≠k.

We allow interventions affect the error variance of the target, but require the effect to be uniform across targets, modeling an experimental intervention with uniform effects on noise.

**Assumption 3.** Let unknown α∈R. Then ∀k, if $Var\left(\epsilon_{k}^{\left(0\right)}\right)=\sigma_{k}^{2}$ then $Var\left(\epsilon_{k}^{\left(k\right)}\right)=\frac{\sigma_{k}^{2}}{\alpha^{2}}$.

Here, α is shared across interventions. As α→∞, this interventional model is equivalent to $do\left(X_{k}=0\right)$.^18^ Under these assumptions, the variance of the target is

$$\text{Var}\left(X_{k}^{\left(k\right)}\right)=\frac{\sigma_{k}^{2}}{\alpha^{2}}\text{.}$$

Let $\hat{\text{Var}}$ denote the unbiased sample variance. We then obtain the estimator

(Equation 4) $${\hat{\Omega }}_{0}=\text{diag}\left(\hat{\text{Var}}\left(X_{1}^{\left(1\right)}\right),\ldots ,\hat{\text{Var}}\left(X_{p}^{\left(p\right)}\right)\right)\text{.}$$

## Results

### dotears

DAGs with NO TEARS^12^ transforms the combinatorial constraint W∈D into the continuous constraint h(W)=0, where ∘ denotes the Hadamard product and

h(W)=tr[exp(W∘W)]−p.

Define $∥\cdot {∥}_{1}$ as the vector $l_{1}$ norm on vec(W), i.e., $∥\text{vec}\left(W\right){∥}_{1}$, and ${\left\|\cdot \right\|}_{F}$ the Frobenius norm. For some loss function F, the differentiability of *h* allows for the optimization framework

(Equation 5) $$\min_{W}\;F\left(W,X\right)+\lambda ∥W{∥}_{1}$$

s.t.h(W)=0.

We present dotears, a consistent, intervention-aware joint estimation procedure for structure learning. Loh and Buhlmann (2014) showed that the Mahalanobis norm is a consistent estimator of $W_{0}$, and is uniquely minimized in expectation by $W_{0}$ given $\Omega_{0}$, but give no estimation procedure for $\Omega_{0}$^16^^,^^19^. Note the Mahalanobis norm’s characterization as inverse-variance-weighted by $\Omega_{0}$,

(Equation 6) $$\|\left(X-XW\right)\Omega_{0}^{-\frac{1}{2}}|{|}_{F}^{2}=\sum_{i=1}^{p}\frac{1}{\sigma_{i}^{2}}\|{\left(X-XW\right)}_{i}|{|}_{F}^{2}\text{.}$$

dotears solves the following optimization problem:

(Equation 7) $$\min_{W}\;\frac{1}{p}\sum_{k=0}^{p}\frac{1}{2n_{k}}\|\left(X^{\left(k\right)}-X^{\left(k\right)}W^{\left(k\right)}\right){\hat{\Omega }}_{0}^{-\frac{1}{2}}|{|}_{F}^{2}+\lambda ∥W{∥}_{1}$$

s.t.h(W)=0,

where

$$W_{ij}^{\left(k\right)}=\left\{ \begin{array}{cc} W_{ij} & j\neq k\\ 0 & j=k \end{array}\right.\text{.}$$

dotears retains the continuous DAG constraint and $l_{1}$ regularization of *W* from NO TEARS,^12^ but incorporates exogenous variance structure through ${\hat{\Omega }}_{0}$ as well as interventional data (k=1,…,p).

#### dotears successfully corrects for exogenous variance structure

We show that dotears is robust to exogenous variance structure, and motivate the necessity of the marginal estimation of ${\hat{\Omega }}_{0}$ using the simplest non-trivial DAG $X_{1}\overset{w}{\to }X_{2}$, where gene $X_{1}$ regulates gene $X_{2}$ with effect size *w*. Assume $X_{1}\overset{w}{\to }X_{2}$ has true weighted adjacency matrix $W_{0}:=\left(\begin{array}{cc} 0 & w\\ 0 & 0 \end{array}\right)$ and SEM

(Equation 8) $$\begin{array}{l} X_{1}=\epsilon_{1},\text{Var}\left(\epsilon_{1}\right)=\sigma_{1}^{2}\text{,}\\ X_{2}=wX_{1}+\epsilon_{2},\text{Var}\left(\epsilon_{2}\right)=\sigma_{2}^{2}\text{.} \end{array}$$

**Figure 1:**
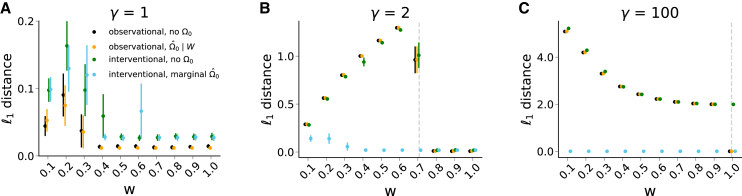
dotears successfully corrects for exogenous variance structure in two-node DAG simulations Comparison of $l_{1}$ distance (lower is better) between true structure and estimates from NO TEARS (black), GOLEM-NV(orange), NO TEARS interventional (green), and dotears (blue). Each method corrects differently for $\Omega_{0}$. For each w=0.1,0.2,…,1.0 and γ=1,2,100, we generate Gaussian data from the structure $X_{1}\overset{w}{\to }X_{2}$ such that $\sigma_{1}^{2}=\gamma \sigma_{2}^{2}$. For each pair w,γ, we draw 25 simulations at a sample size of n=3000. The dashed gray line represents the varsortability bound $\left|w\right|\geq \sqrt{1-\frac{1}{\gamma }}$. a) Under the equal variance assumption γ=1, correction for $\Omega_{0}$ is unneeded. All methods are sufficient for structure recovery. b) At γ=2, NO TEARS (black), GOLEM-NV (orange), and NO TEARS interventional (green) infer correctly on varsortable *w*. c) As γ grows large, the varsortability bound approaches |w|≥1. Only dotears estimates correctly for all *w*. Points represent mean estimates, and bars represent standard errors; some standard errors are too small to see.

Let $\gamma :=\frac{\sigma_{1}^{2}}{\sigma_{2}^{2}}$, such that $\Omega_{0}=\left(\begin{array}{cc} \gamma & 0\\ 0 & 1 \end{array}\right)$ and $W_{0}=\left(\begin{array}{cc} 0 & w\\ 0 & 0 \end{array}\right)$. The least squares loss used by NO TEARS is minimized in expectation if and only if

(Equation 9) $$\left|w\right|\geq \sqrt{1-\frac{1}{\gamma }}\text{,}$$

which is true if and only if the topological ordering of the DAG follows increasing marginal variance, or equivalently a *varsortable* DAG. For the full proof, see Supplementary Material S1.1, or Reisach et al. and Kaiser et al.^15^^,^^19^ In Figure 1, we examine the performance of four different strategies of correcting for $\Omega_{0}$ in simulations. NO TEARS (black) uses the least squares loss, which ignores $\Omega_{0}$, while GOLEM-NV (orange) uses a likelihood loss that estimates ${\hat{\Omega }}_{0}|W$^12^^,^^13^. We also include the scenario when ${\hat{\Omega }}_{0}$ is set to $I_{p}$ in Equation 7. Call this NO TEARS interventional (green), the simplest extension of NO TEARS to interventional data. NO TEARS interventional is aware of hard interventions through the structure $W^{\left(k\right)}$, but ignores $\Omega_{0}$. NO TEARS interventional is thus an ablation study on ${\hat{\Omega }}_{0}$. For each set (w,γ)∈{0.1,…,1.5}×{1,2,4,10,100}, we draw 25 simulations of observational and interventional data, with sample size n=(p+1)∗1000=3000. For observational data, this is 3000 observations from the observational system; for interventional data, this is 1000 observations from each system k=0,1,2. For observational data, we draw Gaussian data under the SEM in Equation 8. For interventional methods, we draw Gaussian data under the system of SEMs in Supplementary Material S1.4. To isolate the behavior of the loss, we remove $l_{1}$ regularization. Full simulation details and results are given in Supplementary Material S1.4. NO TEARS does not correct for $\Omega_{0}$ and uses only observational data. As a result, in Figure 1 it estimates correctly only on varsortable pairs of w,γ. Note that the gray dashed line represents the theoretical varsortability cutoff $\left|w\right|\leq \sqrt{1-\frac{1}{\gamma }}$ given in Equation 9. GOLEM-NV uses the maximum likelihood estimate $\hat{\Omega }|W$ under a Gaussian model. Subsequently, joint estimation is performed over $W,\hat{\Omega }|W$ using the Gaussian negative log likelihood and profile likelihood for $\Omega_{0}$, which simplifies to

(Equation 10) $$\frac{1}{2}\sum_{i=1}^{p}\log\left(\|{\left(X-XW\right)}_{i}|{|}_{F}^{2}\right)\text{.}$$

This profile likelihood is insufficient to correct for exogenous variance structure, and only infers varsortable structures in Figure 1. This evidence qualitatively holds in simulations on three node topologies (Supplementary Material S1.5), where the behavior of GOLEM-NV remains deterministic in w,γ. Joint estimation of $W,{\hat{\Omega }}_{0}|W$ is thus still sensitive to exogenous variance structure. Through NO TEARS interventional, we also see that interventional data alone, without correction for $\Omega_{0}$, is insufficient to infer the two node structure. The NO TEARS interventional estimate is also deterministic in w,γ in Figure 1, and behaves almost identically to NO TEARS and GOLEM. Note that since NO TEARS interventional is given interventional data for all nodes, it operates in a fully identifiable setting (where NO TEARS and GOLEM-NV do not). We also show that CoLiDE-NV, which uses observational data to try to correct for $\Omega_{0}$, is insufficient to infer structure (Figure S1).^20^ Thus, neither interventional data nor correction by ${\hat{\Omega }}_{0}|W$ are alone sufficient to infer structure. dotears combines the two to give the marginal estimate of $\Omega_{0}$ in Equation 4 and a robust estimate of *W*. We do not imply that other methods using interventional data cannot infer the two node case under interventional data; in fact, many do successfully (see Supplementary Material S1.4). Rather, we use **1.)** an observational procedure that ignores $\Omega_{0}$, **2.)** an observational procedure that corrects for an estimated ${\hat{\Omega }}_{0}$, **3.)** an interventional procedure that ignores $\Omega_{0}$, and **4.)** dotears, an interventional procedure that corrects for an estimated ${\hat{\Omega }}_{0}$, to motivate dotears as most parsimonious model robust to exogenous variance structure under this line of thought.

#### Optimization and consistency

We now wish to show that dotears is a consistent estimator of the true DAG. However, two natural problems arise from our usage of ${\hat{\Omega }}_{0}$. First, we have provided no estimation procedure for α, but $E{\hat{\Omega }}_{0}\neq \Omega_{0}$ for α≠1. However, $E{\hat{\Omega }}_{0}\propto \Omega_{0}$ for all α, and constant scalings of Ω are rescalings of the loss.^16^ ${\hat{\Omega }}_{0}$ is therefore well-specified for inference on observational data k=0. However, if α≠1 then ${\hat{\Omega }}_{0}$ is still misspecified for interventional data k≠0. Under Assumption 3

$$\text{Cov}\left(\epsilon^{\left(k\right)}\right)=\text{diag}\left(\sigma_{1}^{2},\sigma_{2}^{2},\ldots ,\frac{\sigma_{k}^{2}}{\alpha^{2}},\ldots ,\sigma_{p}^{2}\right)\text{,}$$

and thus $E{\hat{\Omega }}_{0}\propto \text{Cov}\left(\epsilon^{\left(k\right)}\right)$. A naive approach might estimate ${\hat{\Omega }}_{0}$ from interventional data only and then estimate $\hat{W}$ from observational data only. However, this approach ignores a majority of our data and performs substantially worse in simulations (Supplementary Material S1.6). We show that ${\hat{\Omega }}_{0}$ is well-specified even for k≠0, and the estimation of α is unnecessary (see Corollary 2). Under a sub-Gaussian assumption, Loh and Buhlmann show consistency of the Mahalanobis norm on observational data given $\Omega_{0}$.^16^ We extend these results, using ${\hat{\Omega }}_{0}\overset{p}{\to }\Omega_{0}$, to show

$$\underset{W}{\arg\;\min}\|\left(X^{\left(k\right)}-X^{\left(k\right)}W^{\left(k\right)}\right){\hat{\Omega }}_{0}^{-\frac{1}{2}}|{|}_{F}^{2}$$

is a consistent estimator of $W_{0}^{\left(k\right)}$ for each *k*, where $\overset{p}{\to }$ denotes convergence in probability. A full proof is given in Supplementary Material S1.2.

### Simulations

**Figure 2:**
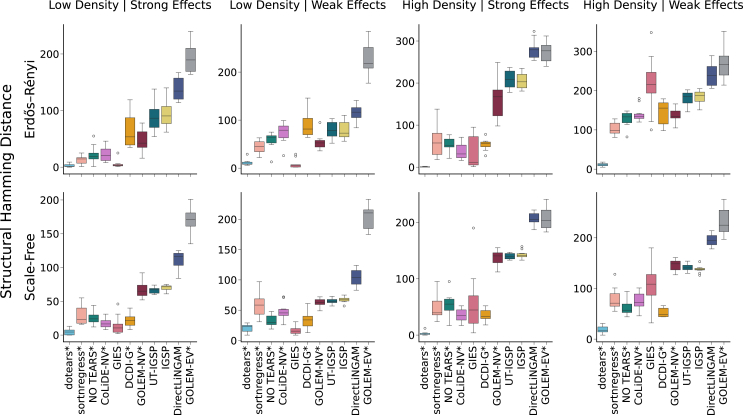
dotears outperforms other methods in large-scale random DAG simulations Method performance on large random graphs (*p* = 40) using Structural Hamming Distance (lower is better) Rows index Erdős-Rényi or Scale Free topologies. Columns index parameterizations of edge density and weight, ordered in increasing difficulty. For details, see STAR Methods. 10 simulations were drawn for each parameterization with sample size (p+1)∗100=4100. ∗ indicates cross-validated methods. Methods are sorted by average performance.

We evaluate structure learning methods across a range of DAG topologies, effects distributions, and generative models. We benchmark methods that leverage interventional data (dotears, GIES, IGSP, UT-IGSP, DCDI) and methods using only observational data (NO TEARS, sortnregress, GOLEM-EV, GOLEM-NV, DirectLiNGAM, CoLiDE-NV).^10^^,^^12^^,^^13^^,^^14^^,^^15^^,^^20^^,^^21^^,^^22^^,^^23^^,^^24^ dotears outperforms all tested methods in DAG estimation and is robust to reasonable violations of the model. Some methods come with important caveats for evaluation. sortnregress is not intended as a “true” structure learning method, but benchmarks the data’s varsortability.^15^ UT-IGSP can infer structure with unknown interventional targets, but we constrain to known targets for fairness.^23^ Most simulations use Gaussian data, but dotears, NO TEARS, sortnregress, GIES, IGSP, and UT-IGSP do not assume Gaussianity. The non-Gaussianity assumption is violated for Direct-LiNGAM, and the equal variance assumption for GOLEM-EV.^13^^,^^24^ We simulate synthetic data from large Erdős-Rényi and Scale-Free DAGs^25^^,^^26^ (p=40), with 10 replicates each. We simulate under four parameterizations: {LowDensity,HighDensity}×{WeakEffects,StrongEffects}. Observational and interventional data have matched sample size n=(p+1)×100=4100. Methods using 5-fold cross-validation for hyperparameter tuning are denoted by ∗. For non-binary methods, edge weights are thresholded. Methods are robust to threshold choice (see STAR Methods).^15^ For simulation, cross-validation, thresholding details, and memory and runtime benchmarking, see STAR Methods. We note that the memory usage of DCDI-G was extreme even with no hidden layers. On average, dotears outperforms all tested methods in structural recovery (Structural Hamming Distance (SHD), Figure 2) and edge weight recovery ($l_{1}$ distance, Figure S9). Furthermore, dotears outperforms all other methods in most parameterizations. GIES matches dotears in “Low Density” simulations, but performs substantially worse in “High Density” simulations. We hypothesize that the greedy nature of GIES hinders performance in more complex DAGs. The primary modeling assumptions of dotears are **1.)** hard interventions (Assumption 1), **2.)** shared α across interventions (Assumption 3), and **3.)** linearity of the SEM. In Supplementary Material S1.7.1 and S1.7.2 we assess the sensitivity of dotears to each assumption. In addition, in Supplementary Material S1.7.1 we also consider **4.)** simulations under different interventional models. We find that dotears performance can change under violations of the hard intervention assumption, but is robust to violations of its interventional model and linearity. Surprisingly, dotears is the second best performing method under a mean-shift intervention model. Moreover, dotears outperforms the neural network method DCDI-DSF in nonlinear simulations, even with “imperfect” interventions, where the main factor determining performance is denseness of the DAG.

### Genome-wide Perturb-seq

**Figure 3:**
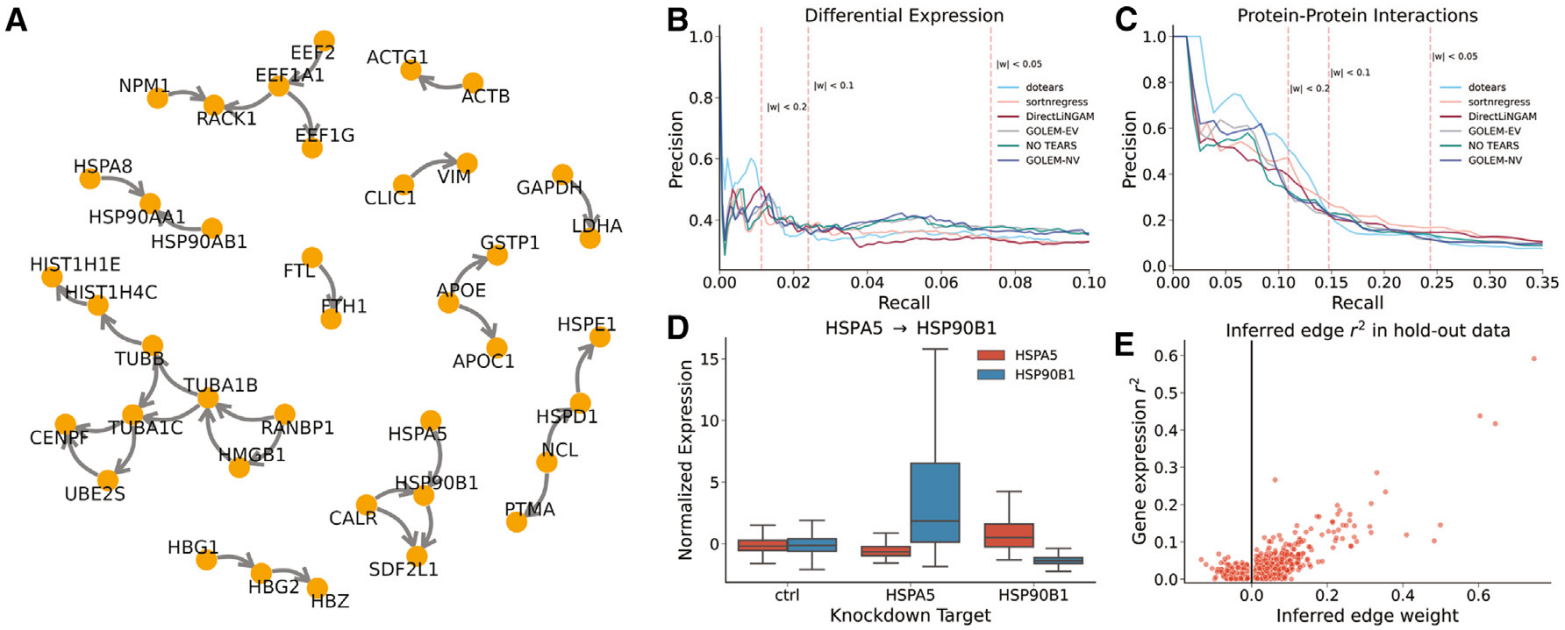
Differential Expression tests and protein-protein interactions validate dotears-inferred regulatory networks in genome-wide Perturb-seq data (A) dotears-inferred network. Edges with magnitude less than 0.2, and genes without inferred edges, were removed (B) Precision-recall curves across differential expression calls made by DESeq2. Dashed red lines indicate recall of dotears at thresholds of |w|<0.2,0.1, and 0.05 respectively. (C) Precision-recall curves across high confidence protein-protein interactions nominated by STRING. Dashed red lines indicate recall of dotears at thresholds of |w|<0.2,0.1, and 0.05 respectively. d) dotears infers HSPA5 → HSP90B1. HSPA5 knockdown increases expression of HSP90B1, but HSP90B1 knockdown does not change HSPA5 expression. (D) dotears inferred edges show correlated gene expression in hold-out observational data.

We apply all benchmarked methods in simulations to a genome-wide Perturb-seq experiment from Replogle et al*.*^5^ We validate inferred edges through **1.)** differential expression tests in the training data using DESeq2^27^^,^^28^ and **2.)** an orthogonal set of high-confidence protein-protein interactions from the STRING database.^29^ We also examine gene-gene correlations in held-out observational expression data. High-confidence edges inferred by dotears show differential expression and/or protein-protein interactions more frequently than those found by other methods, and dotears outperforms all other methods in precision and recall under reasonable thresholding. Replogle et al. provide both normalized and raw data. We select the top 100 most variable genes in the raw observational data. We then benchmark all methods on the normalized, feature-selected data. Cross-validation is not performed due to low sample sizes in some knockdowns; instead, the $l_{1}$ penalty is arbitrarily set to 0.1 for all methods where appropriate. Figure 3A shows the network inferred by dotears, thresholded at |w|<0.2. For full details, see STAR Methods. We use DESeq2 differential expression calls and high-confidence protein-protein interactions from the STRING database to validate inferred edges.^27^^,^^28^^,^^29^ For differential expression, we call an edge i→j a true positive if either gene shows differential expression under knockdown of the other. This is because all methods struggle equally with predicting directionality (see Table S7). For protein-protein interactions, we take high-confidence physical interactions as true positives, where here “high-confidence” is defined by STRING as having a confidence level of over 70%.^29^ Figure 3B and 3C show precision and recall across thresholds for differential expression calls and the protein-protein interactions, respectively. Vertical lines indicate different thresholding regimes. Observational methods were only given observational data; for results when given both observational and interventional data, see STAR Methods. dotears shows much higher precision than all other methods at equivalent recall. Over 65% of inferred edges validated by either differential expression or high-confidence protein-protein interactions. GIES, IGSP, UT-IGSP, and DCDI are excluded because they infer binary edges. These methods inferred 3038, 3064, 3075, and 2039 out of a possible 4950 edges, respectively; no other method predicted more than 700 even at a weight threshold of 0.05. Accordingly, they had almost random precision - see STAR Methods and Tables S4–S6 for detailed results for all methods at multiple thresholds. These results reinforce concerns about the scalability of GIES to more complex scenarios. For DCDI, we report intermediate results, since convergence was not obtained under 24 h on GPU training. CPU training attempts ran out of memory even after the allocation of 180GB; GPU training attempts also repeatedly ran out of memory. We re-run dotears on the same data excluding the observational data, and use the held-out observational data to validate inferred edges in the knockdown data. For each pair of genes i,j, Figure 3E shows the inferred edge weight against the $r^{2}$ of i,j in the observational data. Note that we take the largest magnitude weight between $w_{ij}$ and $w_{ji}$. Figure 3E shows a clear relationship between inferred edge weight in knockdown data and the $r^{2}$ in observational expression data.

## Discussion

We present dotears, a structure learning framework that uses interventional data to estimate exogenous variance structure and subsequently leverages observational and interventional data to learn the causal graph. We showed that dotears is appropriate for Peturb-seq data analysis and can recover high-confidence gene regulation events and show that dotears outperforms all tested state-of-the-art methods in simulations. Finally, we prove that the loss function used by dotears provides consistent DAG estimation under mild assumptions. In simulations, simple methods generally outperform complex methods in structure recovery. In particular, dotears and sortnregress regularly outperform more complex methods, including neural network methods, even under modeling assumption violations and nonlinear data. Their strong performance also shows the effectiveness of using variance patterns to infer structure. In real data, dotears infers gene regulation events supported by knockdown expression in training data, orthogonal high-confidence protein-protein interactions, and gene expression correlations in held out observational data. In general, dotears provides robust inference of relevant gene regulatory events. We show that dotears is robust to error variance structure or model misspecification. Furthermore, dotears-inferred edges validate with higher precision than any other method without sacrificing power. The relatively high precision of dotears-inferred regulatory events provides confidence in identifying targets for potential experimental validation.

### Limitations of the study

One limitation of dotears not addressed in simulations is that it assumes that every node has a corresponding intervention in order to estimate the error variance. Without intervention on every node, it is difficult to properly specify $\Omega_{0}$.

## Resource availability

### Lead contact

Requests for further information and resources should be directed to and will be fulfilled by the lead contact, Albert Xue (asxue@ucla.edu).

### Materials availability

This study did not generate new unique reagents.

### Data and code availability

• This article analyzes existing, publicly available data, i.e., the genome-wide Perturb-seq described in Replogle et al.^5^ and accessible at the link in the key resources table.
• All original code has been deposited at https://github.com/asxue/dotears/and is publicly available at https://doi.org/10.5281/zenodo.14286216 as of the date of publication.
• Any additional information required to reanalyze the data reported in this article is available from the lead contact upon request.

## Acknowledgments

AX was supported by the 10.13039/100000002NIH Training Grant in Genomic Analysis and Interpretation T32HG002536. This work was partially funded by HHMI Hanna H Gray and Sloan fellows programs to HP. This work was partially funded by 10.13039/100000002NIH grant (R35GM125055) and NSF grants (CAREER-1943497, IIS-2106908) to SS. We thank Nathan LaPierre for helpful conversations.

## Author contributions

Conceptualization, A.X., S.S., and H.P.; methodology, A.X., S.S., and H.P.; formal analysis, A.X., J.R., S.S., and H.P.; investigation, A.X., S.S., and H.P.; data curation, A.X.; writing – original draft, A.X.; writing – review and editing, A.X., J.R., S.S., and H.P.; funding acquisition, A.X., S.S., and H.P.; supervision, S.S. and H.P.

## Declaration of interests

The authors declare no competing interests.

## STAR★Methods

### Key resources table

**Table:** 

REAGENT or RESOURCE	SOURCE	IDENTIFIER
**Deposited data**		

Genome-scale Perturb-seq experiment data	Replogle et al.^5^	https://plus.figshare.com/articles/dataset/_Mapping_information-rich_genotype-phenotype_landscapes_with_genome-scale_Perturb-seq_Replogle_et_al_2022_processed_Perturb-seq_datasets/20029387
STRING protein-protein interactions database	Szklarczyk et al.^29^	https://string-db.org/

**Software and algorithms**		

DAGs with NO TEARS	Zheng et al.^12^	https://github.com/xunzheng/notears
Python 3.9	Python Software Foundation	https://www.python.org/
R 4.1.0	R Software	https://www.r-project.org/
DESeq2	Ahlmann-Eltze and Huber.^28^	https://bioconductor.org/packages/release/bioc/html/DESeq2.html
Snakemake	Köster and Rahmann^30^	https://snakemake.readthedocs.io/en/stable/

### Method details

#### Large random graph simulations

##### Data generation

For evaluation, data is generated for DAGs with p=40 nodes. Structures are drawn from both Erdős-Rényi (ER) and Scale-Free (SF) DAGs.^25^^,^^26^ We parameterize ER graphs as ER-*r* and SF graphs as SF-*z*, where r∈[0,1] represents the probability of assignment of each individual edge, whereas z∈Z is the integer number of edges assigned per node. We simulate under two parameterizations {LowDensity,HighDensity} of the edge densities (r,z). In the “Low Density” parameterization, we give (r,z)=(0.1,2). To evaluate performance on higher density topologies, we also give “High Density” parameterizations, where (r,z)=(0.2,4). Given an edge density scenario, we simulate under two parameterizations of the edge weights. In the “Strong Effects” parameterization, w∼Unif([−2.0,−0.5]∪[0.5,2.0]). We also give the “Weak Effects” parameterization, which edge weights are drawn from w∼Unif([−1.0,0.3]∪[0.3,1.0]) such that |w|≤1 is guaranteed. Here, there is no guarantee any two nodes will be varsortable (although they may be in practice, as a function of $\Omega_{0}$).^15^^,^^19^ Table S1 summarizes all four possible simulation parameterizations. For each node *i*, we draw $\sigma_{i}∼\text{Unif}\left(\left[0.5,2.0\right]\right)$, and draw $n_{0}$ observations from $\epsilon_{i}^{\left(0\right)}∼N\left(0,\sigma_{i}^{2}\right)$. For each DAG we generate an instance of observational data, where $n_{0}=\left(p+1\right)*n_{k}=4100$, and an instance of interventional data, where $n_{k}=100$ for all k=0…p to match sample size. We set the distribution of $\epsilon_{i}^{\left(k\right)}$ according to Assumptions 3 and 2 for α=4. For dotears, NO TEARS, sortnregress, GOLEM-EV, GOLEM-NV, CoLiDE-NV and DCDI-G 5-fold cross-validation was performed to select the regularization parameters. For each drawn DAG, a separate data instance of interventional and observational data was re-drawn from the same distribution specifically for cross-validation. After choosing a λ (or for GOLEM-EV and GOLEM-NV, the set $\left(\lambda_{1},\lambda_{2}\right)$) from the data for cross-validation, the methods were evaluated on the original simulated data. For dotears, NO TEARS, sortnregress, and DCDI-G, 5-fold cross-validation was performed across the grid λ∈{.001,.01,.1,1,10,100}. For GOLEM-EV and GOLEM-NV, 5-fold cross-validation was performed across the grid $\lambda_{1}\times \lambda_{2}\in \left\{.001,.01,.1,1,10,100\right\}\times \left\{.05,.5,5,50\right\}$. We threshold at 0.2 for “Weak Effects” simulations, and at 0.3 for “Strong Effects” simulations. Methods are generally robust to thresholding choice. Results for precision and recall on thresholded edges are shown in Figures S7 and S8, respectively.

##### Benchmarking

In Figure S5, we benchmark wallclock time and memory usage for all methods in p=40 simulations^30^ on the UCLA hoffman2 cluster. All continuous optimization methods (dotears, NO TEARS, GOLEM-EV, GOLEM-NV, and CoLiDE-NV) have significantly higher average runtimes than other methods, which is partially explained by cross-validation procedures. dotears has relatively light memory usage, outperformed only by NO TEARS, sortnregress, and CoLiDE-NV. DCDI-G has enormous memory requirements, especially relative to other methods. We note that for DCDI-G, the reported benchmarks do not include memory usage or runtime from cross-validation folds. Instead, we report only the “main” run of DCDI-G, and thus DCDI-G is not denoted with the ∗ indicating cross-validation.

##### Thresholding on large simulation results

Edge thresholding for weighted adjacency matrices is necessary for accurate evaluation using SHD, but the choice of threshold can feel arbitrary. We find that methods are generally robust to thresholding choice, following similar results from Reisach et al*.*^15^ Figure S6 examines the effect on thresholding of small weights in *W* in large random DAG simulations, for methods that infer weighted adjacency matrices (dotears, NO TEARS, sortnregress, DirectLiNGAM, GOLEM-NV, GOLEM-EV, and CoLiDE-NV).^12^^,^^13^^,^^15^^,^^24^ For simplicity, we summarize simulations in terms of the generative edge distribution. Simulations with “Weak Effects”, w∼Unif([−1.0,−0.3]∪[0.3,1.0]), are shown in Figure S6a; simulations with “Strong Effects”, w∼Unif([−2.0,−0.5]∪[0.5,2.0]) are shown in Figure S6b. We compare the SHD between the ground truth adjacency matrix and the inferred adjacency matrix for each method at all thresholds between 0 and the absolute lower bound of the true edge weight distribution (0.3 for “Weak Effects”, 0.5 for “Strong Effects”). Any edge whose magnitude is below the chosen threshold is set to 0 for SHD evaluation. Figure S6 shows that thresholding is necessary for the evaluation of SHD between weighted adjacency matrices, but also that methods are generally robust to thresholding choice. Without thresholding (equivalently, a threshold of 0), SHD results are inflated, but recover even at low thresholds.

##### Edge weight estimation for large random simulations

Accurate estimation of edge weights is important for understanding structure. Figure 2 gives results on structural recovery through SHD, but does not inform edge weight recovery. To measure edge weight recovery we use $l_{1}$ distance, defined as the vector $l_{1}$ norm between the flattened true weighted adjacency matrix and the flattened inferred weighted adjacency matrix. $l_{1}$ distance gives information on both structure recovery and edge weight estimation simultaneously. In Figure S9, we benchmark the recovery of edge weights for methods that return a weighted adjacency matrix (dotears, sortnregress, NO TEARS, GOLEM-EV, GOLEM-NV, DirectLiNGAM, CoLiDE-NV).^12^^,^^13^^,^^15^^,^^20^^,^^24^ For fairness, we exclude methods that only return a binary adjacency matrix. Methods are thresholded in the same manner as in Figure 2 dotears outperforms all other methods in terms of effect size recovery. In addition, the relative ordering of the methods stays consistent with Figure 2.

### Quantification and statistical analysis

In simulations, we benchmark in terms of Structural Hamming Distance (SHD), the number of edge additions, removals, or reversals needed to change one DAG into another. Figures either depict mean (point) and standard error (bar) (Figure 1) or boxplot quartiles (Figures 2 and 3). Details of statistical tests can be found in the following sections.

#### Statistical significance of SHD distribution difference

For each method, and across all large random graph simulations, we compare the SHD distributions, marginalized across all simulation parameterizations, against that of dotears. For each method, we perform a one-sided Mann-Whitney U test to test difference between the method’s SHD distribution and the SHD distribution of dotears. Here, n=80 is the total number of large random graph simulations. The resulting *p*-values are reported in Table S2, and are significant for all methods.

#### Genome-wide Perturb-seq

We benchmark all methods on a single-cell Perturb-seq experiment from Replogle et al.*,*^5^ who provide both raw count data as well as normalized data, where normalized here means a z-scoring relative to the mean and standard deviation of the control dataset, accounting for batch. Here, the control dataset indicates a set of pre-selected control cells, and not simply cells with non-targeting guides. For details, see Replogle et al*.*^5^ We perform feature selection in the raw data. We select the top 100 most variable genes in the raw observational data, excluding **1.)** genes coding for ribosomal proteins and **2.)** genes without knockdown data. Here, the observational data indicates cells incorporating non-targeting control guides. We then take the normalized expression data for these selected 100 genes in **1.)** the observational data, and **2.)** each of the 100 knockdowns. This forms our training set in Figures 3A–3D. In Figure 3E, we perform the same procedure as above, but exclude **1.)** the observational data. In other words, our training set is formed exclusively from expression data in the 100 knockdowns. Cross-validation is not performed due to low sample sizes in some knockdowns; instead, the L1 penalty is arbitrarily set to 0.1 for all methods where appropriate. Otherwise, method settings are the same as simulations. For DCDI, we report intermediate results, since convergence was not obtained under 24 h even on GPU training. CPU training was attempted with 30 cores and 180 GB memory, but was killed by the operating system. GPU training was attempted, but was also repeatedly killed by the operating system for memory constraints. DCDI is run as DCDI-G, since DCDI-DSF would not fit in memory, and is also run with imperfect interventions and known interventions. DCDI reported 2982 total edges, but many of these are both causal and anti-causal; in total, 2039 “interactions” were reported.

#### Differential expression testing

We use DESeq2 to test for differential expression.^27^^,^^28^ We calculate size factors across the raw feature-selected data, and use these in all downstream tests. Next, for each knockdown ko(i), we test for differential expression for all genes *j* compared to the observational data. For single-cell data, we run DESeq with parameters test = LRT, fitType = glmGamPoi, useT = TRUE, minmu = 1e-6, minReplicatesForReplace = Inf, reduced = 1. We repeat this for each knockdown independently in turn. Here, *n* is the number of cells in each knockdown, and can be found in Replogle et al.. At the end of this procedure, we have $100^{2}$ unadjusted *p*-values. We perform Benjamini-Hochberg correction to an FDR level of 0.05. Subsequently, in ko(i), if gene *j* has an adjusted *p*-value less than 0.05, we call that a true edge in downstream calculations of precision and recall.

#### Protein-protein interactions

As a second validation edge set, we use protein-protein interactions from the STRING database.^29^ We pull protein-protein interactions for Homo Sapiens down, and use only physical evidence for interactions. Protein-protein interactions were only used if the STRING confidence score was over 70%.

#### Precision recall tables

We present precision and recall at three edge threshold levels (|w|>0.2, |w|<0.1, and |w|<0.05) for all methods. We use both differential expression calls and protein-protein interactions as true sets, and separate results for both. Tables S4, S5, and A6 denote results for |w|>0.2, |w|<0.1, and |w|<0.05, respectively. For differential expression calls we ignore causal direction, and instead for two given genes denote any differential expression in either direction as a “true” edge. This is because all methods struggle equally with inferring causal direction under differential expression, and call an equal number of edges in the “correct” causal direction as the “incorrect” anticausal direction (see Table S7).

#### Inclusion of interventional data for observational methods

In Figure 3, observational methods (sortnregress, DirectLiNGAM, GOLEM-EV, GOLEM-NV, NO TEARS, CoLiDE-NV) are only given observational data. It is reasonable to wonder if their decreased performance relative to dotears is due to a substantial sample size decrease (93691 total to 75328 purely observational). To test this, we also ran the observational methods on observational and interventional data concatenated. Since these are observational methods, there is no way to label these data as interventional; instead, these methods assume they are observational. Results are shown in Figure S21. No substantial relative performance difference was observed in the new trials. All observational methods still perform worse than dotears, especially in high-confidence regimes.

### Experimental model and study participant details

This study did not include experiments with a specific model or subject.

## Supplemental information

Document S1. Figures S1–S21 and Tables S1–S7

Data S1. Contains proofs of method consistency and varsortability bounds
