# Supplementary material for: dotears: Scalable and consistent directed acyclic graph estimation using observational and interventional data
Source: iScience. 2024 Dec 24;28(2):111673. doi: 10.1016/j.isci.2024.111673 (PMC11814701; doi:10.1016/j.isci.2024.111673)
Supplement: Document S1. Figures S1–S21 and Tables S1–S7 [file mmc1.pdf]

iScience, Volume *28*

Supplemental information

dotears: Scalable and consistent directed acyclic graph estimation using observational and interventional data

Albert Xue, Jingyou Rao, Sriram Sankararaman, and Harold Pimentel

S1. Supplementary Material

S1.1. Least squares infers varsortable structures in expectation

We first derive an explicit cutoff for varsortability, matching the cutoff given in Eq. 9.

Lemma 1. *Let* $\gamma := \frac{\sigma_1^2}{\sigma_2^2}$. *The system* $X_1 \overset{w}{\to} X_2$ *is varsortable if and only if* $|w| \geq \sqrt{1 - \frac{1}{\gamma}}$.

For the proof, see Supplementary Material S1.1.1. We verify that the least squares loss infers varsortable structures by comparing the SEM in Eq. 8 against the false but Markov equivalent model $X_1 \overset{\delta}{\leftarrow} X_2$, with weighted adjacency matrix $W_\delta := \begin{pmatrix} 0 & 0 \\ \delta & 0 \end{pmatrix}$ and SEM

$$\begin{aligned} X_1 &= \delta X_2 + \epsilon_1, \\ X_2 &= \epsilon_2. \end{aligned} \tag{11}$$

Let $(\cdot)_i$ represent the ith column vector, and denote the least squares loss as

$$\ell\left(W, \boldsymbol{X}\right) := \frac{1}{2n_0} \sum_{i=1}^{p} ||(\boldsymbol{X} - \boldsymbol{X}W)_i||_F^2 \,. \tag{12}$$

Theorem 1. *Let* $\gamma := \frac{\sigma_1^2}{\sigma_2^2}$. $\mathbb{E}\ell\left(W_0, \boldsymbol{X}\right) \leq \mathbb{E}\ell\left(W_\delta, \boldsymbol{X}\right)$ *for all* δ *if and only if* $|w| \geq \sqrt{1 - \frac{1}{\gamma}}$.

Corollary 1. *In the system* $X_1 \overset{w}{\to} X_2$*, the least squares loss is uniquely minimized in expectation by the true DAG if and only if the system is varsortable.*

For the proof of Theorem 1, see Supplementary Material S1.1.2.

S1.1.1. Proof of Lemma 1

Proof. Under the generative SEM in Eq. 8, we have

$$\begin{aligned} \operatorname{Var}\left(X_1\right) &= \sigma_1^2 \\ \operatorname{Var}\left(X_2\right) &= w^2\sigma_1^2 + \sigma_2^2 \end{aligned} . \tag{13}$$

The system is therefore varsortable if and only if $w^2\sigma_1^2 + \sigma_2^2 \geq \sigma_1^2$. Using the substitution $\sigma_1^2 = \gamma\sigma_2^2$, we obtain

$$\begin{aligned} w^2\sigma_1^2 + \sigma_2^2 &\geq \sigma_1^2 \\ w^2\gamma\sigma_2^2 + \sigma_2^2 &\geq \gamma\sigma_2^2 \\ w^2\gamma + 1 &\geq \gamma \\ |w| &\geq \sqrt{1 - \frac{1}{\gamma}} \end{aligned} \tag{14}$$

□

S1.1.2. Proof of Theorem 1

Proof. From Eq. 12, we can calculate both $\mathbb{E}\ell\left(W_0, \boldsymbol{X}\right)$ and $\mathbb{E}\ell\left(W_\delta, \boldsymbol{X}\right)$ component-wise. Under W_0 we obtain component-wise

$$\begin{aligned} \left(X - XW_0\right)_1 &= X_1 \\ &= \epsilon_1 && \text{by Eq. 8} \\ \left(X - XW_0\right)_2 &= X_2 - wX_1 \\ &= \epsilon_2 && \text{by Eq. 8} \end{aligned} \tag{15}$$

As a result,

$$\begin{aligned}\mathbb{E}\ell\left(W_0, \boldsymbol{X}\right) &= \frac{1}{2n_0}\sum_{i=1}^{p}\mathbb{E}\left|\left|\left(\boldsymbol{X}-\boldsymbol{X}W_0\right)_i\right|\right|_F^2 \\ &= \frac{1}{2n_0}\left(\mathbb{E}\left(\left|\left|\boldsymbol{\epsilon}_1\right|\right|_F^2\right)+\mathbb{E}\left(\left|\left|\boldsymbol{\epsilon}_2\right|\right|_F^2\right)\right) \\ &= \frac{1}{2}\left(\sigma_1^2+\sigma_2^2\right)\end{aligned}$$

Here we note that the expected loss under the generative DAG is $\mathbb{E}\ell\left(W_0, \boldsymbol{X}\right) = \frac{1}{2}\sum_{i=1}^{p}\text{Var}\left(\epsilon_i\right)$.

Similarly, under W_δ we have

$$\begin{aligned}\left(X - XW_\delta\right)_1 &= X_1 - \delta X_2 \\ &= \epsilon_1 - \delta\left(w\epsilon_1+\epsilon_2\right) \quad \text{by Eq. 8} \\ &= (1-\delta w)\epsilon_1 - \delta\epsilon_2 \\ \left(X - XW_\delta\right)_2 &= X_2 \\ &= w\epsilon_1 + \epsilon_2 \qquad\qquad \text{by Eq. 8}\end{aligned} \tag{16}$$

and therefore $\mathbb{E}\ell\left(W_\delta, \boldsymbol{X}\right)$ is

$$\frac{1}{2}\left(\sigma_1^2\left(w^2+(1-\delta w)^2\right)+\sigma_2^2\left(1+\delta^2\right)\right).$$

We can now ask when $\mathbb{E}\ell\left(W_\delta, \boldsymbol{X}\right) \leq \mathbb{E}\ell\left(W_0, \boldsymbol{X}\right)$, or equivalently,

$$\begin{aligned}\sigma_1^2\left(w^2+(1-\delta w)^2\right)+\sigma_2^2\left(1+\delta^2\right) &\leq \sigma_1^2+\sigma_2^2 \\ \gamma\sigma_2^2\left(w^2+(1-\delta w)^2\right)+\sigma_2^2\left(1+\delta^2\right) &\leq \gamma\sigma_2^2+\sigma_2^2 \\ \gamma\left(w^2+1-2\delta w+\delta^2 w^2\right)+1+\delta^2 &\leq \gamma+1 \\ \gamma\left(w^2-2\delta w+\delta^2 w^2\right)+\delta^2 &\leq 0 \\ \delta^2\left(1+\gamma w^2\right)-\delta\left(2\gamma w\right)+\gamma w^2 &\leq 0\end{aligned} \tag{17}$$

The inequality becomes a quadratic in δ, which when solved gives us roots at

$$\begin{aligned}\delta &= \frac{2\gamma w \pm \sqrt{(-2\gamma w)^2 - 4\left(1+\gamma w^2\right)\left(\gamma w^2\right)}}{2\left(1+\gamma w^2\right)} \\ &= \frac{2\gamma w \pm 2\gamma w\sqrt{1-\frac{1}{\gamma}\left(1+\gamma w^2\right)}}{2\left(1+\gamma w^2\right)} \\ &= \frac{\gamma w\left(1\pm\sqrt{1-w^2-\frac{1}{\gamma}}\right)}{1+\gamma w^2}.\end{aligned} \tag{18}$$

Note that for the quadratic in Eq. 17, $\gamma w^2 \geq 0$. Then $\exists\delta$ such that $\mathbb{E}\ell\left(W_\delta, \boldsymbol{X}\right) \leq \mathbb{E}\ell\left(W_0, \boldsymbol{X}\right)$ when the term in the root exists in Eq. 18. Equivalently, we can say that $\mathbb{E}\ell\left(W_0, \boldsymbol{X}\right) \leq \mathbb{E}\ell\left(W_\delta, \boldsymbol{X}\right)$ is guaranteed for all δ if and only if

$$|w| \geq \sqrt{1-\frac{1}{\gamma}}. \tag{19}$$

□

S1.2. Proof of Consistency

For a diagonal matrix Ω, define

$$\begin{aligned}\mathcal{L}_\Omega\left(W^{(k)}, \boldsymbol{X}^{(k)}\right) &:= \frac{1}{n_k}\left|\left|\left(\boldsymbol{X}^{(k)}-\boldsymbol{X}^{(k)}W^{(k)}\right)\Omega^{-\frac{1}{2}}\right|\right|_F^2 \\ &= \frac{1}{n_k}\sum_{j=1}^{p}\frac{1}{\Omega_{jj}}\left|\left|\left(\boldsymbol{X}^{(k)}-\boldsymbol{X}^{(k)}W^{(k)}\right)_j\right|\right|_F^2,\end{aligned} \tag{20}$$

where Ω_{jj} is the j,jth entry of Ω. Further, recall the definition

$$\Omega_0^{(k)} := \mathrm{Cov}\left(\epsilon^{(k)}\right).$$

Denote convergence in probability by $\xrightarrow{p}$. We show that under a sub-Gaussian assumption, the `dotears` loss is a consistent estimator of the true DAG.

Assumption 4. *For any k, we assume $X^{(k)}$ is a sub-Gaussian random vector with parameter σ^2.*

Lemma 2. *For any intervention k,*

$$\mathcal{L}_{\Omega_0}\left(W^{(k)}, \boldsymbol{X}^{(k)}\right) = \mathcal{L}_{\Omega_0^{(k)}}\left(W^{(k)}, \boldsymbol{X}^{(k)}\right) + \mathcal{O}_W(1)$$

For the proof, see Supplementary Material S1.2.1.

Corollary 2.

$$\arg\min_W \mathcal{L}_{\Omega_0}\left(W^{(k)}, \boldsymbol{X}^{(k)}\right) = \arg\min_W \mathcal{L}_{\Omega_0^{(k)}}\left(W^{(k)}, \boldsymbol{X}^{(k)}\right)$$

For any intervention k and any α, Lemma 2 and Corollary 2 show that true exogenous variance structure $\Omega_0 = \alpha^2 \mathbb{E}\hat{\Omega}_0$ is sufficient for structure recovery. Results from Loh and Buhlmann (2014) are then sufficient to establish consistency of the estimator $\arg\min_W \mathcal{L}_{\Omega_0}\left(W^{(k)}, X^{(k)}\right)$. We wish to show consistency of $\arg\min_W \mathcal{L}_{\hat{\Omega}_0}\left(W^{(k)}, \boldsymbol{X}^{(k)}\right)$, where we have an estimated $\hat{\Omega}_0$ rather than Ω_0.

For simplicity, in the following we assume $\alpha = 1$ without loss of generality, which is justified by Corollary 2 and Lemma 2. Note then that $\Omega_0 = \Omega_0^{(k)}$, which allows us to drop the ${}^{(k)}$ notation.

Remark 1. *For consistency under an estimated $\hat{\Omega}_0$, we have two cases: the observational case $k = 0$ and the interventional case $k \neq 0$. The observational case is simplest - in particular, when $k = 0$, $\hat{\Omega}_0$ is independent of $\boldsymbol{X}^{(0)}$, and we may therefore freely estimate $W_0^{(0)}$ from $\mathcal{L}_{\hat{\Omega}_0}\left(W^{(0)}, \boldsymbol{X}^{(0)}\right)$. When $k \neq 0$, we can assume independence of $\hat{\Omega}_0$ and $\boldsymbol{X}^{(k)}$ under a data splitting framework, where some fraction of our n_k samples are reserved for estimation of $\hat{\sigma}_k^2$, and the remaining samples are given in $\boldsymbol{X}^{(k)}$.*

Assumption 5. *Let*

$$\hat{\sigma}_k^2 := \left(\hat{\Omega}_0\right)_{kk}.$$

Then

$$\hat{\sigma}_k^2 \perp\!\!\!\perp \boldsymbol{X}_k^{(k)}.$$

However, in the presented applications we use the full n_k samples of $\boldsymbol{X}_k^{(k)}$ for both estimates.

Note that we abuse notation to write $\boldsymbol{X}^{(k)}$ as the set of samples used to estimate $\hat{W}_0^{(k)}$ given $\hat{\Omega}_0$. Further, for convenience we abuse notation to write n_k as the sample size of $\boldsymbol{X}^{(k)}$ AND the sample size of $\hat{\sigma}_k^2$, since the respective sample sizes are equal to n_k up to a multiplicative constant which can be ignored as $n_k \to \infty$.

We further define

$$\mathbb{E}\mathcal{L}_{\Omega}\left(W^{(k)}, \boldsymbol{X}^{(k)}\right) := \frac{1}{n_k}\sum_{j=1}^{p} \frac{1}{\Omega_{jj}} \mathbb{E}\left|\left|\left(\boldsymbol{X}^{(k)} - \boldsymbol{X}^{(k)} W^{(k)}\right)_j\right|\right|_F^2.$$

Here, we abuse notation to remove Ω_{jj} from the expectation. Moreover, for the random vector X we write

$$\mathbb{E}\mathcal{L}_{\Omega}\left(W^{(k)}, X^{(k)}\right) := \sum_{j=1}^{p} \frac{1}{\Omega_{jj}} \mathbb{E}\left[\left(X^{(k)} - X^{(k)} W^{(k)}\right)_j^2\right],$$

and note that the two definitions are equivalent under expectation. Note further that

$$\mathrm{score}_{\Omega}(W) \equiv \mathbb{E}\mathcal{L}_{\Omega}(W, X),$$

where score is defined in Loh and Buhlmann.[16]

Remark 2. *Loh and Buhlmann differentiate between the* ***weighted adjacency matrix*** *W and the* ***binary*** *DAG G. In particular, they define*

$$score_{\Omega}\left(G, X\right) := \min_{W \in \mathcal{U}_G} \left\{score_{\Omega}\left(W, X\right)\right\},$$

where $\mathcal{U}_G$ is the set of weighted adjacency matrices with support in G. However, for the true binary DAG G_0,

$$score_{\Omega}\left(G, X\right) = score_{\Omega}\left(G_0, X\right) \quad \forall G \supseteq G_0,$$

which leads to identifiability issues (see Lemma 6 and discussion of Lemma 19[16]).

We define everything directly on the weighted adjacency matrix W, and ignore the binary DAG G. This discrepancy is essentially due to methodological differences. Loh and Buhlmann first find a superset of the binary structure G_0, and subsequently rely on sparse regression to recover edge weights.[16] However, `dotears` *searches directly in the space of weighted adjacency matrices to find W_0. While the end result is similar, we use this remark to explain discrepancies in notation and proof structure for readers following with Loh and Buhlmann.[16]*

We start by restating results from Loh and Buhlmann.[16] Let $\Omega_1 \in \mathbb{R}^{p\times p}$ an arbitrary diagonal weight matrix. Define

$$a_{\max} := \lambda_{\max}\left(\Omega_0\Omega_1^{-1}\right),\ a_{\min} := \lambda_{\min}\left(\Omega_0\Omega_1^{-1}\right)$$

to be the maximum and minimum ratios between the corresponding diagonal entries of Ω_0 and Ω_1, and further define

$$\xi_{\Omega}^{(k)} := \min_{\substack{W\in\mathcal{D}\\ W\neq W_0}} \mathbb{E}\mathcal{L}_{\Omega}\left(W^{(k)}, X^{(k)}\right) - \mathbb{E}\mathcal{L}_{\Omega}\left(W_0^{(k)}, X^{(k)}\right) \tag{21}$$

to be the additive gap between the expected loss of the true weighted adjacency matrix W_0 and the expected loss of the next-best weighted adjacency matrix under an arbitrary diagonal matrix Ω. Note that $\xi_{\Omega_0} > 0$ (see Theorem 7 in Loh and Buhlmann[16]). Then we have the following theorem, whose proof is given in Loh and Buhlmann:[16]

Theorem 2. *Suppose*

$$\frac{a_{max}}{a_{min}} \leq 1 + \frac{\xi_{\Omega_0}^{(k)}}{p}. \tag{22}$$

Then $W_0^{(k)} \in \arg\min_{W\in\mathcal{D}}\left\{\mathbb{E}\mathcal{L}_{\Omega_1}\left(W, X^{(k)}\right)\right\}$. If Inequality 22 is strict, then $W_0^{(k)}$ is the unique minimizer of $\mathbb{E}\mathcal{L}_{\Omega_1}\left(W, X^{(k)}\right)$.

Theorem 3. *For any intervention k, as $n_j \to \infty$ for all $j = 1, \ldots, p$, with high probability $W_0^{(k)}$ is the unique minimizer of $\mathbb{E}\mathcal{L}_{\hat{\Omega}_0}\left(W^{(k)}, X^{(k)}\right)$, and thus $\xi_{\hat{\Omega}_0}^{(k)} > 0$.*

For the proof, see Supplementary Material S1.2.2.

Lemma 3. *For any intervention k, suppose $X^{(k)}$ is a sub-Gaussian random vector with parameter σ^2. Then $X_k^{(k)}$ is a sub-Gaussian random variable with parameter σ^2, and for all $t \geq 0$, we have*

$$\left|\frac{1}{\hat{\sigma}_k^2} - \frac{1}{\sigma_k^2}\right| \leq \frac{2\sigma^2}{\sigma_k^2}\cdot\max\{\delta, \delta^2\}$$

with probability at least $1 - 2\exp(-cn_k t^2)$, where $\delta = c'\sqrt{\frac{1}{n_k}} + c''t$ and

$$\frac{\sigma^2}{\sigma_k^2}\cdot\max\{\delta, \delta^2\} \leq \frac{1}{2}. \tag{23}$$

Proof. Lemma 3 is a re-statement of Lemma 29 in Loh and Buhlmann[16] (Appendix E) for the degenerate case $p = 1$. □

Following Loh and Buhlmann,[16] we introduce the new notation $f^{(k)}_{\sigma_j}$. For a node j and a set $S \subseteq \{1 \ldots p\} \backslash \{j\}$, define

$$f^{(k)}_{\sigma_j}(S) \coloneqq \frac{1}{\sigma_j^2}\mathbb{E}\left[\left(X_j^{(k)} - X_S^{(k)} w_j\right)^2\right],$$

where $X_j^{(k)}$ is the jth column vector of $X^{(k)}$, and $X_S^{(k)} w_j$ is the best linear predictor for $X_j^{(k)}$ regressed upon $X_S^{(k)}$. Similarly,

$$\hat{f}^{(k)}_{\sigma_j}(S) \coloneqq \frac{1}{\sigma_j^2}\frac{1}{n_k}\left|\left|\boldsymbol{X}_j^{(k)} - \boldsymbol{X}_S^{(k)}\hat{w}_j\right|\right|_F^2,$$

where $\hat{w}_j$ is the ordinary least squares solution for linear regression of $\boldsymbol{X}_j^{(k)}$ upon $\boldsymbol{X}_S^{(k)}$, i.e.

$$\begin{aligned}
\hat{w}_j &\coloneqq \left(\left(\left(\boldsymbol{X}_S^{(k)}\right)^{\mathrm{T}}\boldsymbol{X}_S^{(k)}\right)^{-1}\left(\boldsymbol{X}_S^{(k)}\right)^{\mathrm{T}}\boldsymbol{X}_j^{(k)}\right) \\
&= \left(\left(\left(\boldsymbol{X}_S^{(k)}\right)^{\mathrm{T}}\boldsymbol{X}_S^{(k)}\right)^{-1}\left(\boldsymbol{X}_S^{(k)}\right)^{\mathrm{T}}\left(\boldsymbol{X}_S^{(k)} w_j + \boldsymbol{e}_j^{(k)}\right)\right) \\
&= w_j + \left(\left(\boldsymbol{X}_S^{(k)}\right)^{\mathrm{T}}\boldsymbol{X}_S^{(k)}\right)^{-1}\left(\boldsymbol{X}_S^{(k)}\right)^{\mathrm{T}}\boldsymbol{e}_j^{(k)},
\end{aligned} \tag{24}$$

where note that

$$\boldsymbol{X}_j^{(k)} = \boldsymbol{X}_S^{(k)} w_j + \boldsymbol{e}_j^{(k)}.$$

Lastly, denote the vector ℓ_2 norm as $|||\cdot|||_2$.

Loh and Buhlmann achieve a high-dimensional consistency result by first conditioning on the support of a sparse precision matrix $\Theta^{(k)}$, since the support of $\Theta^{(k)} \coloneqq \mathrm{Cov}\left(X^{(k)}\right)^{-1}$ defines the *moralized* graph, an undirected graph obtained from a DAG by adding edges between all parents with a shared child node.[16,31] Since the edge set of the moralized graph is a superset of the edge set of the true DAG, for a given node j one may condition on the maximum size of the putative neighbor set, $N_{\Theta^{(k)}}(j)$. Indeed, in Loh and Buhlmann,[16] Loh and Buhlmann restrict $|N_{\Theta^{(k)}}(j)| \leq d$ for all j, with the only restriction on d being that $d \leq n$.

`dotears` does not condition on $\Theta^{(k)}$, and therefore does not condition on the moralized graph or $N_{\Theta}(j)$. To maintain the validity of our consistency proof, we let $d = p - 1 \leq n$. Under this restriction, we no longer have a high-dimensional result, but maintain consistency of the loss function of `dotears` for low dimensionality of p.

Assumption 6. *For all $k = 0, \ldots, p$, let $d = p - 1 \leq n_k$.*

Lemma 4. *For any intervention k, suppose $X^{(k)}$ is sub-Gaussian with parameter σ^2. Then $\forall j$ and $S \subseteq \{1 \ldots p\} \backslash \{j\}$,*

$$\sigma_j^2\left|\hat{f}^{(k)}_{\hat{\sigma}_j}(S) - \hat{f}^{(k)}_{\sigma_j}(S)\right| \leq \sigma^2 \cdot \max\{\delta, \delta^2\} \cdot \frac{C}{n_k}\left|\left|\boldsymbol{e}_j^{(k)}\right|\right|_2^2 \tag{25}$$

with probability $1 - c_1\exp(-c_2\log p)$, where $\delta = c'\sqrt{\frac{1}{n_j}} + c''\sqrt{\frac{\log p}{n_j}}$, if

$$\frac{\sigma^2}{\sigma_j^2} \cdot \delta \leq \frac{1}{2}. \tag{26}$$

For the proof, see Supplementary Material S1.2.3.

Lemma 5. *For any intervention k, suppose $X^{(k)}$ is sub-Gaussian with parameter σ^2. Then as $n_i \to \infty$ for all $i = 1 \ldots p$, $\forall j$ and $S \subseteq \{1 \ldots p\} \backslash \{j\}$ it is true that*

$$\left|\hat{f}^{(k)}_{\hat{\sigma}_j}(S) - f^{(k)}_{\sigma_j}(S)\right| \leq c_0\sigma^4\sqrt{\frac{\log p}{n_j}} + c_1\sigma^2\sqrt{\frac{\log p}{n_k}} + c_2\frac{p}{n_k} \tag{27}$$

with probability at least $1 - c_1\exp(-c_2\log p)$.

For the proof, see Supplementary Material S1.2.4.

Theorem 4. *For any intervention k, suppose Inequality 27 holds, and suppose*

$$\left(c_0\sigma^4\sqrt{\frac{\log p}{n_j}}+c_1\sigma^2\sqrt{\frac{\log p}{n_k}}+c_2\frac{p}{n_k}\right)\cdot\sum_{j=1}^{p}\frac{1}{\sigma_j^2}<\frac{\xi_{\Omega_0}^{(k)}}{2}. \tag{28}$$

Then

$$\mathcal{L}_{\hat{\Omega}_0}\left(W_0^{(k)},\boldsymbol{X}^{(k)}\right)<\mathcal{L}_{\hat{\Omega}_0}\left(W^{(k)},\boldsymbol{X}^{(k)}\right)$$

$\forall W\in\mathcal{D}$, and the estimator

$$\hat{W}^{(k)}:=\arg\min_{W}\mathcal{L}_{\hat{\Omega}_0}\left(W,\boldsymbol{X}^{(k)}\right) \tag{29}$$

is therefore consistent as $n_j\to\infty$ for all $j=1\ldots p$.

For the proof, see Supplementary Material S1.2.5.

S1.2.1. Proof of Lemma 2

Proof. Note that

$$\begin{aligned}
\mathcal{L}_{\Omega_0^{(k)}}\left(W^{(k)},\boldsymbol{X}^{(k)}\right):=&\frac{1}{n_k}\left|\left|\left(\boldsymbol{X}^{(k)}-\boldsymbol{X}^{(k)}W^{(k)}\right)\left(\Omega_0^{(k)}\right)^{-\frac{1}{2}}\right|\right|_F^2\\
=&\frac{1}{n_k}\sum_{i=1}^{p}\frac{1}{\mathrm{Var}\left(\epsilon_i^{(k)}\right)}\left|\left|\left(\boldsymbol{X}^{(k)}-\boldsymbol{X}^{(k)}W^{(k)}\right)_i\right|\right|_F^2\\
=&\frac{1}{n_k}\frac{\alpha^2}{\sigma_k^2}\left|\left|\left(\boldsymbol{X}^{(k)}-\boldsymbol{X}^{(k)}W^{(k)}\right)_k\right|\right|_F^2+\frac{1}{n_k}\sum_{\substack{i=1\\i\neq k}}^{p}\frac{1}{\sigma_i^2}\left|\left|\left(\boldsymbol{X}^{(k)}-\boldsymbol{X}^{(k)}W^{(k)}\right)_i\right|\right|_F^2\\
\mathcal{L}_{\Omega_0}\left(W^{(k)},\boldsymbol{X}^{(k)}\right):=&\frac{1}{n_k}\left|\left|\left(\boldsymbol{X}^{(k)}-\boldsymbol{X}^{(k)}W^{(k)}\right)\Omega_0^{-\frac{1}{2}}\right|\right|_F^2\\
=&\frac{1}{n_k}\sum_{i=1}^{p}\frac{1}{\sigma_i^2}\left|\left|\left(\boldsymbol{X}^{(k)}-\boldsymbol{X}^{(k)}W^{(k)}\right)_i\right|\right|_F^2.
\end{aligned}$$

Let $I_0^k=\mathrm{diag}(1,1,\ldots,0,\ldots,1)$ a modifier on the $p\times p$ identity matrix, where the diagonal entries are all 1 except for the k,k entry, which is 0.

Then in the interventional system,

$$W^{(k)}=WI_0^k$$

and thus

$$\mathcal{L}_{\Omega_0}\left(W^{(k)},\boldsymbol{X}^{(k)}\right)=\frac{1}{n_k}\left|\left|\left(\boldsymbol{X}^{(k)}-\boldsymbol{X}^{(k)}WI_0^k\right)\Omega_0^{-\frac{1}{2}}\right|\right|_F^2$$

We expand $\mathcal{L}_{\Omega_0}$ to obtain

$$\begin{aligned}
\mathcal{L}_{\Omega_0}\left(W^{(k)},\boldsymbol{X}^{(k)}\right)=&\frac{1}{n_k}\sum_{i=1}^{p}\frac{1}{\sigma_i^2}\left|\left|\left(\boldsymbol{X}^{(k)}-\boldsymbol{X}^{(k)}W^{(k)}\right)_i\right|\right|_F^2\\
=&\frac{1}{n_k}\frac{1}{\sigma_k^2}\left|\left|\left(\boldsymbol{X}^{(k)}-\boldsymbol{X}^{(k)}W^{(k)}\right)_k\right|\right|_F^2+\frac{1}{n_k}\sum_{\substack{i=1\\i\neq k}}^{p}\frac{1}{\sigma_i^2}\left|\left|\left(\boldsymbol{X}^{(k)}-\boldsymbol{X}^{(k)}W^{(k)}\right)_i\right|\right|_F^2
\end{aligned}$$

and note that

$$\begin{aligned}
\left(\boldsymbol{X}^{(k)}W^{(k)}\right)_k&=\boldsymbol{X}^{(k)}\left(WI_0^k\right)_k\\
&=\boldsymbol{X}^{(k)}\vec{0}_p\\
&=\vec{0}_{n_k}.
\end{aligned}$$

As a result,

$$\left|\left|\left(\boldsymbol{X}^{(k)}-\boldsymbol{X}^{(k)}W^{(k)}\right)_k\right|\right|_F^2=\left|\left|\boldsymbol{X}_k^{(k)}\right|\right|_F^2=\mathcal{O}_W(1)$$

is constant in W, and

$$\begin{aligned}\mathcal{L}_{\Omega_0}\left(W^{(k)},\boldsymbol{X}^{(k)}\right)=&\frac{1}{n_k}\frac{1}{\sigma_k^2}\left|\left|\left(\boldsymbol{X}^{(k)}-\boldsymbol{X}^{(k)}W^{(k)}\right)_k\right|\right|_F^2+\frac{1}{n_k}\sum_{\substack{i=1\\i\neq k}}^{p}\frac{1}{\sigma_i^2}\left|\left|\left(\boldsymbol{X}^{(k)}-\boldsymbol{X}^{(k)}W^{(k)}\right)_i\right|\right|_F^2\\=&\frac{1}{n_k}\frac{1}{\sigma_k^2}\left|\left|\boldsymbol{X}_k^{(k)}\right|\right|_F^2+\frac{1}{n_k}\sum_{\substack{i=1\\i\neq k}}^{p}\frac{1}{\sigma_i^2}\left|\left|\left(\boldsymbol{X}^{(k)}-\boldsymbol{X}^{(k)}W^{(k)}\right)_i\right|\right|_F^2\\=&\mathcal{L}_{\Omega_0^{(k)}}\left(W^{(k)},\boldsymbol{X}^{(k)}\right)+\mathcal{O}_W(1)\end{aligned}$$

□

S1.2.2. Proof of Theorem 3

Proof. Let $a_{\max}:=\lambda_{\max}\left(\Omega_0\hat{\Omega}_0^{-1}\right)$, and $a_{\min}:=\lambda_{\min}\left(\Omega_0\hat{\Omega}_0^{-1}\right)$ similarly. We first prove that $a_{\max}\xrightarrow{p}\alpha^2$ without loss of generality. For all k, note first that

$$\frac{\sigma_k^2}{\hat{\sigma}_k^2}\xrightarrow{p}\alpha^2.$$

Then by Continuous Mapping Theorem we have

$$a_{\max}\xrightarrow{p}\alpha^2.$$

Similarly, $a_{\min}\xrightarrow{p}\alpha^2$, and therefore

$$\frac{a_{\max}}{a_{\min}}\xrightarrow{p}1.$$

We note that $\frac{a_{\max}}{a_{\min}}=1$ is almost impossible empirically, but can be controlled with high probability to 1 with arbitrary precision. Then by Theorem 2, with high probability $W_0^{(k)}$ is the unique minimizer of $\mathbb{E}\mathcal{L}_{\hat{\Omega}_0}\left(W^{(k)},X^{(k)}\right)$ as $n_j\to\infty$ for all $j=1\ldots p$. Further, $\xi_{\hat{\Omega}_0}^{(k)}>0$. □

S1.2.3. Proof of Lemma 4

Proof. Let the projection matrix of $\boldsymbol{X}_S^{(k)}$ be defined as $P_{\boldsymbol{X}_S^{(k)}}:=\boldsymbol{X}_S^{(k)}\left(\left(\boldsymbol{X}_S^{(k)}\right)^{\mathrm{T}}\boldsymbol{X}_S^{(k)}\right)^{-1}\left(\boldsymbol{X}_S^{(k)}\right)^{\mathrm{T}}$.

Given the least squares estimate in Eq. 24, we may write

$$\begin{aligned}\sigma_j^2\cdot\hat{f}_{\sigma_j}^{(k)}(S)&=\frac{1}{n_k}\left|\left|\boldsymbol{X}_j^{(k)}-\boldsymbol{X}_S^{(k)}\hat{w}_j\right|\right|_F^2\\&=\frac{1}{n_k}\left|\left|\boldsymbol{X}_j^{(k)}\left(w_j-\hat{w}_j\right)+\boldsymbol{e}_j^{(k)}\right|\right|_F^2\\&=\frac{1}{n_k}\left|\left|\left(I-P_{\boldsymbol{X}_S^{(k)}}\right)\boldsymbol{e}_j^{(k)}\right|\right|_F^2.\end{aligned}\tag{30}$$

Note by Triangle Inequality, we have for the ℓ_2 norm $||\cdot||_2$ and the spectral norm $|||\cdot|||_2$

$$\begin{aligned}\left|\left|\left|\left(I-P_{\boldsymbol{X}_S^{(k)}}\right)\boldsymbol{e}_j^{(k)}\right|\right|_F-\left|\left|\boldsymbol{e}_j^{(k)}\right|\right|_2\right|&\leq\left|\left|P_{\boldsymbol{X}_S^{(k)}}\boldsymbol{e}_j^{(k)}\right|\right|_F\\&\leq\left|\left|\left|P_{\boldsymbol{X}_S^{(k)}}\right|\right|\right|_2\left|\left|\boldsymbol{e}_j^{(k)}\right|\right|_2\\&\leq\left|\left|\boldsymbol{e}_j^{(k)}\right|\right|_2,\end{aligned}$$

where the spectral norm of the projection matrix $\left|\left|\left|P_{\boldsymbol{X}_S^{(k)}}\right|\right|\right|_2$ is 1. Then

$$\sigma_j^2 \cdot \hat{f}_{\sigma_j}^{(k)}(S) = \frac{1}{n_k}\left|\left|\boldsymbol{X}_j^{(k)} - \boldsymbol{X}_S^{(k)}\hat{w}_j\right|\right|_F^2 \leq \frac{2}{n_k}\left|\left|\boldsymbol{e}_j^{(k)}\right|\right|_2^2.$$

Expanding the left hand side of Eq. 25, we obtain

$$\begin{aligned}
\sigma_j^2\left|\hat{f}_{\hat{\sigma}_j}^{(k)}(S) - \hat{f}_{\sigma_j}^{(k)}(S)\right| =& \sigma_j^2\left|\frac{1}{\hat{\sigma}_j^2} - \frac{1}{\sigma_j^2}\right|\frac{1}{n_k}\left|\left|\boldsymbol{X}_j^{(k)} - \boldsymbol{X}_S^{(k)}\hat{w}_j\right|\right|_F^2 \\
\leq& \sigma_j^2 \cdot 2\frac{\sigma^2}{\sigma_j^2}\cdot \max\{\delta, \delta^2\} \cdot \frac{1}{n_k}\left|\left|\boldsymbol{X}_j^{(k)} - \boldsymbol{X}_S^{(k)}\hat{w}_j\right|\right|_F^2 \\
\leq& 2\sigma^2 \cdot \max\{\delta, \delta^2\} \cdot \frac{2}{n_k}\left|\left|\boldsymbol{e}_j^{(k)}\right|\right|_2^2
\end{aligned}$$

with probability $\min\{1 - c_1\exp(-c_2\log p), 1 - 2\exp(-cn_jt^2)\}$, where $\delta = c'\sqrt{\frac{1}{n_j}} + c''t$, if $\frac{\sigma^2}{\sigma_j^2}\cdot\max\{\delta, \delta^2\} \leq \frac{1}{2}$. The first inequality is by Lemma 3. Set $t = \sqrt{\frac{\log p}{n_j}}$. Then

$$\begin{aligned}
\delta &= c'\sqrt{\frac{1}{n_j}} + c''\sqrt{\frac{\log p}{n_j}}, \\
\delta^2 &= (c')^2\frac{1}{n_j} + (c'')^2\frac{\log p}{n_j} + 2c'c''\frac{\sqrt{\log p}}{n_j}.
\end{aligned}$$

By Assumption 6, $\log p < p - 1 < n_j$. Then $\max\{\delta, \delta^2\} = \delta$, and $1 - 2\exp(-cn_jt^2) = 1 - 2\exp(-c\log p)$. □

S1.2.4. Proof of Lemma 5

Proof. Give the singular value decomposition on the n_k by p matrix $\boldsymbol{X}_S^{(k)}$ as

$$\boldsymbol{X}_S^{(k)} = U\Sigma V^{\mathrm{T}},$$

where U is an $n_k \times p$ matrix, Σ is a diagonal $p \times p$ matrix, V is a $p \times p$ matrix, and $U^{\mathrm{T}}U = V^{\mathrm{T}}V = I$. Then

$$\begin{aligned}
\boldsymbol{X}_S^{(k)}\left(\left(\boldsymbol{X}_S^{(k)}\right)^{\mathrm{T}}\boldsymbol{X}_S^{(k)}\right)^{-1}\left(\boldsymbol{X}_S^{(k)}\right)^{\mathrm{T}} =& U\Sigma V^{\mathrm{T}}\left(V\Sigma^2V^{\mathrm{T}}\right)^{-1}V\Sigma U^{\mathrm{T}} \\
=& UU^{\mathrm{T}}.
\end{aligned}$$

We substitute into the expansion in Equation 30 to obtain

$$\begin{aligned}
\sigma_j^2 \cdot \hat{f}_{\sigma_j}^{(k)}(S) =& \frac{1}{n_k}\left|\left|\left(I - \boldsymbol{X}_S^{(k)}\left(\left(\boldsymbol{X}_S^{(k)}\right)^{\mathrm{T}}\boldsymbol{X}_S^{(k)}\right)^{-1}\left(\boldsymbol{X}_S^{(k)}\right)^{\mathrm{T}}\right)\boldsymbol{e}_j^{(k)}\right|\right|_F^2 \\
=& \frac{1}{n_k}\left|\left|\left(I - UU^{\mathrm{T}}\right)\boldsymbol{e}_j^{(k)}\right|\right|_F^2 \\
=& \frac{1}{n_k}\mathrm{tr}\left[\left(\boldsymbol{\epsilon}_j^{(k)}\right)^{\mathrm{T}}\left(I - UU^{\mathrm{T}}\right)\left(I - UU^{\mathrm{T}}\right)\left(\boldsymbol{e}_j^{(k)}\right)\right] \\
=& \frac{1}{n_k}\mathrm{tr}\left[\left(\boldsymbol{e}_j^{(k)}\right)^{\mathrm{T}}\left(I - UU^{\mathrm{T}}\right)\left(\boldsymbol{e}_j^{(k)}\right)\right] \\
=& \frac{1}{n_k}\mathrm{tr}\left[\left(\boldsymbol{e}_j^{(k)}\right)^{\mathrm{T}}\left(\boldsymbol{e}_j^{(k)}\right) - \left(U^{\mathrm{T}}\left(\boldsymbol{e}_j^{(k)}\right)\right)^{\mathrm{T}}U^{\mathrm{T}}\left(\boldsymbol{e}_j^{(k)}\right)\right].
\end{aligned}$$

Let

$$Y := U^{\mathrm{T}}\boldsymbol{e}_j^{(k)} \in \mathbb{R}^p.$$

Then

$$\sigma_j^2 \cdot \hat{f}_{\sigma_j}^{(k)}(S) = \frac{1}{n_k} \mathrm{tr}\left[\left(\boldsymbol{e}_j^{(k)}\right)^{\mathrm{T}}\left(\boldsymbol{e}_j^{(k)}\right) - Y^{\mathrm{T}}Y\right]$$
$$= \frac{1}{n_k}\left[\sum_{i=1}^{n_k}\left(\boldsymbol{e}_{i,j}^{(k)}\right)^2 - \sum_{l=1}^{p} Y_l^2\right].$$

Here, Y is a random vector with expectation 0 and covariance

$$\begin{aligned}\mathrm{Cov}(Y) &= \mathbb{E}(YY^{\mathrm{T}}) \\ &= \mathbb{E}\left[U^{\mathrm{T}}\left(\boldsymbol{e}_j^{(k)}\right)\left(\boldsymbol{e}_j^{(k)}\right)^{\mathrm{T}} U\right] \\ &= U^{\mathrm{T}}\mathbb{E}\left[\left(\boldsymbol{e}_j^{(k)}\right)\left(\boldsymbol{e}_j^{(k)}\right)^{\mathrm{T}}\right] U \\ &= U^{\mathrm{T}}\left(\sigma_j^2 I_{n_k}\right) U \\ &= \sigma_j^2 U^{\mathrm{T}} U \\ &= \sigma_j^2 I_p.\end{aligned}$$

As a result, for fixed p, $\sum_{l=1}^{p} Y_l^2 = \mathcal{O}_p(1)$, and as $n_k \to \infty$ we have $\frac{1}{n_k}\sum_{l=1}^{p} Y_l^2 = \mathcal{O}_p(1) \xrightarrow{p} 0$. Further, since $e_{i,j}^{(k)}$ are i.i.d sub-Gaussian with parameter at most $c\sigma^2$, for $t \geq 0$ we can apply the sub-Gaussian tail bound

$$\mathbb{P}\left(\frac{1}{n_k}\left|\sum_{i=1}^{n_k}\left(\boldsymbol{e}_{i,j}^{(k)}\right)^2 - \mathbb{E}\left[\left(e_{i,j}^{(k)}\right)^2\right]\right| \geq c\sigma^2 t\right) \leq c_1 \exp\left(-c_2 n_k t^2\right).$$

Note that

$$\frac{1}{n_k}\mathbb{E}\left[\left(e_{i,j}^{(k)}\right)^2\right] = \sigma_j^2 \cdot f_{\sigma_j}^{(k)}(S)$$

By setting $t = \sqrt{\frac{\log p}{n_k}}$, we therefore obtain the bound

$$\sigma_j^2\left|\hat{f}_{\sigma_j}^{(k)}(S) - f_{\sigma_j}^{(k)}(S)\right| \leq c\sigma^2\sqrt{\frac{\log p}{n_k}} + \left|\frac{1}{n_k}\sum_{j=1}^{p} Y_j^2\right|$$

with probability at least $c_1 \exp\left(-c_2 \log p\right)$.

We now use Lemma 4. We set $t' = \sqrt{\frac{\log p}{n_j}}$, which for $\delta = c'\sqrt{\frac{1}{n_j}} + c''t'$ gives

$$\begin{aligned}\sigma_j^2\left|\hat{f}_{\hat{\sigma}_j}^{(k)}(S) - f_{\sigma_j}^{(k)}(S)\right| &\leq \sigma_j^2\left(\left|\hat{f}_{\hat{\sigma}_j}^{(k)}(S) - \hat{f}_{\sigma_j}^{(k)}(S)\right| + \left|\hat{f}_{\sigma_j}^{(k)}(S) - f_{\sigma_j}^{(k)}(S)\right|\right) \\ &\leq \sigma^2 \cdot \max\{\delta, \delta^2\} \cdot \frac{C}{n_k}\mathbb{E}\left[\left|\left|\boldsymbol{e}_j^{(k)}\right|\right|_2^2\right] \\ &\quad + c_0\sigma^2\sqrt{\frac{\log p}{n_k}} + \left|\frac{1}{n_k}\sum_{j=1}^{p} Y_j^2\right| \\ &\leq c_0'\sigma^4\sqrt{\frac{\log p}{n_j}} + c_1'\sigma^2\sqrt{\frac{\log p}{n_k}} + c_2'\frac{p}{n_k}\end{aligned}$$

if

$$\frac{\sigma^2}{\sigma_j^2} \cdot \delta \leq \frac{1}{2}.$$

with probability at least $1 - c_1 \exp(-c_2 \log p)$. □

S1.2.5. Proof of Theorem 4

Proof. Suppose the gap $\xi^{(k)}_{\hat{\Omega}_0}$ is nonzero, which we guarantee with high probability by Theorem 3. Then the following inequality is valid, by Eq. 27 and Eq. 28:

$$\begin{aligned}\left|\mathcal{L}_{\hat{\Omega}_0}\left(W^{(k)}, \boldsymbol{X}^{(k)}\right) - \mathbb{E}\mathcal{L}_{\Omega_0}\left(W^{(k)}, X^{(k)}\right)\right| &\leq \sum_{j=1}^{p}\left|\hat{f}^{(k)}_{\hat{\sigma}_j}\left(\{1\ldots p\}\backslash\{j\}\right) - f_{\sigma_j}\left(\{1\ldots p\}\backslash\{j\}\right)\right| \\ &< \frac{\xi^{(k)}_{\hat{\Omega}_0}}{2}\end{aligned} \tag{31}$$

for all $W \in \mathcal{D}$. Then for all $W \in \mathcal{D}$, $W \neq W_0$,

$$\begin{aligned}\mathcal{L}_{\hat{\Omega}_0}\left(W_0^{(k)}, \boldsymbol{X}^{(k)}\right) &< \mathbb{E}\mathcal{L}_{\Omega_0}\left(W_0^{(k)}, X^{(k)}\right) + \frac{\xi^{(k)}_{\hat{\Omega}_0}}{2} \\ &\leq \left(\mathbb{E}\mathcal{L}_{\Omega_0}\left(W^{(k)}, X^{(k)}\right) - \xi^{(k)}_{\Omega_0}\right) + \frac{\xi^{(k)}_{\hat{\Omega}_0}}{2} \\ &< \mathcal{L}_{\hat{\Omega}_0}\left(W^{(k)}, \boldsymbol{X}^{(k)}\right),\end{aligned}$$

where the first and third inequalities come from Eq. 31 and the second inequality comes from the definition of the gap $\xi^{(k)}_{\Omega_0}$. □

S1.3. Two node system - `dotears`

We return to the two-node system described by Eq. 8, and re-examine the system under the `dotears` loss $\mathcal{L}_{\Omega_0}$, defined in Eq. 20. As before, we define the ground-truth weighted adjacency matrix $W_0 := \begin{pmatrix} 0 & w \\ 0 & 0 \end{pmatrix}$ and false weighted adjacency matrix $W_\delta := \begin{pmatrix} 0 & 0 \\ \delta & 0 \end{pmatrix}$. For simplicity, we assume we are given $\Omega_\alpha = \mathbb{E}\hat{\Omega}_0 = \alpha^2\Omega_0$, the expected value of the estimator $\hat{\Omega}_0$. We show that $\mathbb{E}\mathcal{L}_{\Omega_\alpha}\left(W_0^{(k)}, \boldsymbol{X}^{(k)}\right) < \mathbb{E}\mathcal{L}_{\Omega_\alpha}\left(W_\delta^{(k)}, \boldsymbol{X}^{(k)}\right)$ for all $k = 0, 1, 2$.

S1.3.1. Observational system

In the observational system, we retain the generative SEM in Eq. 8:

$$\begin{aligned}X_1^{(0)} &= \epsilon_1^{(0)} \\ X_2^{(0)} &= wX_1^{(0)} + \epsilon_2^{(0)} \\ &= w\epsilon_1^{(0)} + \epsilon_2^{(0)}.\end{aligned} \tag{32}$$

To calculate $\mathbb{E}\mathcal{L}_{\Omega_\alpha}\left(W_0^{(0)}, \boldsymbol{X}^{(0)}\right)$, we decompose the loss $\mathcal{L}$ component-wise by noting that

$$\mathbb{E}\mathcal{L}_{\Omega_\alpha}\left(W^{(k)}, \boldsymbol{X}^{(k)}\right) = \frac{1}{n_k}\sum_{i=0}^{p}\frac{\alpha^2}{\sigma_i^2}\mathbb{E}\left|\left|\left(\boldsymbol{X}^{(k)} - \boldsymbol{X}^{(k)}W^{(k)}\right)_i\right|\right|_F^2,$$

to obtain

$$\begin{aligned}\left(\boldsymbol{X}^{(0)} - \boldsymbol{X}^{(0)}W_0\right)_1 &= \boldsymbol{X}_1^{(0)} \\ &= \boldsymbol{\epsilon}_1^{(0)} \\ \left(\boldsymbol{X}^{(0)} - \boldsymbol{X}^{(0)}W_0\right)_2 &= \boldsymbol{X}_2^{(0)} - w\boldsymbol{X}_1^{(0)} \\ &= w\boldsymbol{\epsilon}_1^{(0)} + \boldsymbol{\epsilon}_2^{(0)} - w\boldsymbol{\epsilon}_1^{(0)} \\ &= \boldsymbol{\epsilon}_2^{(0)}\end{aligned}$$

and therefore

$$\begin{aligned}
\frac{1}{n_0}\frac{\alpha^2}{\sigma_1^2}\mathbb{E}\left|\left|\left(\boldsymbol{X}^{(0)} - \boldsymbol{X}^{(0)}W_0\right)_1\right|\right|_F^2 &= \frac{\alpha^2}{\sigma_1^2}\left(\frac{1}{n_0}\mathbb{E}\left|\left|\boldsymbol{\epsilon}_1^{(0)}\right|\right|_F^2\right) \\
&= \left(\frac{\alpha^2}{\sigma_1^2}\right)\sigma_1^2 \\
&= \alpha^2 \\
\frac{1}{n_0}\frac{\alpha^2}{\sigma_2^2}\mathbb{E}\left|\left|\left(\boldsymbol{X}^{(0)} - \boldsymbol{X}^{(0)}W_0\right)_2\right|\right|_F^2 &= \frac{\alpha^2}{\sigma_2^2}\left(\frac{1}{n_0}\mathbb{E}\left|\left|\boldsymbol{\epsilon}_2^{(0)}\right|\right|_F^2\right) \\
&= \left(\frac{\alpha^2}{\sigma_2^2}\right)\sigma_2^2 \\
&= \alpha^2
\end{aligned}$$

As a result,

$$\mathbb{E}\mathcal{L}_{\Omega_\alpha}\left(W_0^{(0)}, \boldsymbol{X}^{(0)}\right) = 2a^2.$$

Similarly, we calculate $\mathbb{E}\mathcal{L}_{\Omega_\alpha}\left(W_\delta^{(0)}, \boldsymbol{X}^{(0)}\right)$ component-wise:

$$\begin{aligned}
\left(\boldsymbol{X}^{(0)} - \boldsymbol{X}^{(0)}W_\delta\right)_1 &= \boldsymbol{X}_1^{(0)} - \delta\boldsymbol{X}_2^{(0)} \\
&= \boldsymbol{\epsilon}_1^{(0)} - \delta\left(w\boldsymbol{\epsilon}_1^{(0)} + \boldsymbol{\epsilon}_2^{(0)}\right) \\
&= (1-\delta w)\,\boldsymbol{\epsilon}_1^{(0)} - \delta\boldsymbol{\epsilon}_2^{(0)} \\
\left(\boldsymbol{X}^{(0)} - \boldsymbol{X}^{(0)}W_\delta\right)_2 &= \boldsymbol{X}_2^{(0)} \\
&= w\boldsymbol{\epsilon}_1^{(0)} + \boldsymbol{\epsilon}_2^{(0)}
\end{aligned}$$

Let $\gamma \in \mathbb{R}^+$, such that $\sigma_1^2 = \gamma\sigma_2^2$. Then we calculate expected loss component-wise as

$$\begin{aligned}
\frac{1}{n_0}\frac{\alpha^2}{\sigma_1^2}\mathbb{E}\left|\left|\left(\boldsymbol{X}^{(0)} - \boldsymbol{X}^{(0)}W_\delta\right)_1\right|\right|_F^2 &= \frac{\alpha^2}{\sigma_1^2}\left((1-\delta w)^2\sigma_1^2 + \delta^2\sigma_2^2\right) \\
&= \alpha^2\left((1-\delta w)^2 + \frac{\delta^2}{\gamma}\right) \\
\frac{1}{n_0}\frac{\alpha^2}{\sigma_2^2}\mathbb{E}\left|\left|\left(\boldsymbol{X}^{(0)} - \boldsymbol{X}^{(0)}W_\delta\right)_2\right|\right|_F^2 &= \frac{\alpha^2}{\sigma_2^2}\left(w^2\sigma_1^2 + \sigma_2^2\right) \\
&= \alpha^2\left(1 + w^2\gamma\right)
\end{aligned}$$

As a result,

$$\mathbb{E}\mathcal{L}_{\Omega_\alpha}\left(W_\delta^{(0)}, \boldsymbol{X}^{(0)}\right) = \alpha^2\left((1-\delta w)^2 + \frac{\delta^2}{\gamma} + 1 + w^2\gamma\right).$$

We can now ask whether $\mathbb{E}\mathcal{L}_{\Omega_\alpha}\left(W_\delta^{(0)}, \boldsymbol{X}^{(0)}\right) \geq \mathbb{E}\mathcal{L}_{\Omega_\alpha}\left(W_0^{(0)}, \boldsymbol{X}^{(0)}\right)$ for all w, δ, γ.

$$\begin{aligned}
\mathbb{E}\mathcal{L}_{\Omega_\alpha}\left(W_\delta^{(0)}, \boldsymbol{X}^{(0)}\right) &\overset{?}{\geq} \mathbb{E}\mathcal{L}_{\Omega_\alpha}\left(W_0^{(0)}, \boldsymbol{X}^{(0)}\right) \\
\alpha^2\left((1-\delta w)^2 + \frac{\delta^2}{\gamma} + 1 + w^2\gamma\right) &\overset{?}{\geq} 2\alpha^2 \\
(1-\delta w)^2 + \frac{\delta^2}{\gamma} + 1 + w^2\gamma &\overset{?}{\geq} 2 \\
1 - 2\delta w + \delta^2 w^2 + \frac{\delta^2}{\gamma} + w^2 &\overset{?}{\geq} 1
\end{aligned}$$

In the end, we obtain the inequality

$$w^2\left(\delta^2+\gamma\right)-w(2\delta)+\frac{\delta^2}{\gamma}\overset{?}{\geq}0 \tag{33}$$

which is also a quadratic in w. We can solve for the roots of w:

$$\begin{aligned}w&=\frac{2\delta\pm\sqrt{4\delta^2-4\left(\frac{\delta^2}{\gamma}\right)\left(\delta^2+\gamma\right)}}{2\left(\delta^2+\gamma\right)}\\&=\frac{2\delta\pm2\sqrt{\delta^2-\frac{\delta^4}{\gamma}-\delta^2}}{2\left(\delta^2+\gamma\right)}\\&=\frac{2\delta\pm2\sqrt{-\frac{\delta^4}{\gamma}}}{2\left(\delta^2+\gamma\right)}\end{aligned}$$

$\gamma\in\mathbb{R}^+$ shows that w has no solution in $\mathbb{R}$. Moreover, the intercept term $\frac{\delta^2}{\gamma}>0$ in Eq. 33, proving the result for all w,δ,γ.

S1.3.2. Intervention on node 1

We proceed similarly for the interventional cases. Upon intervention on node 1, the generative structure does not change, i.e. $W_0^{(1)}=\begin{pmatrix}0&w\\0&0\end{pmatrix}$, with SEM

$$\begin{aligned}X_1^{(1)}&=\epsilon_1^{(1)}\\X_2^{(1)}&=wX_1^{(1)}+\epsilon_2^{(1)}.\\&=w\epsilon_1^{(1)}+\epsilon_2^{(1)}\end{aligned}$$

Note that $\mathrm{Var}\left(\epsilon_i^{(1)}\right)=\frac{\sigma_1^2}{\alpha^2}=\frac{1}{\alpha^2}\mathrm{Var}\left(\epsilon_i^{(0)}\right)$, in accordance with Assumption 3. Component-wise under $W_0^{(1)}$ we have

$$\begin{aligned}\left(\boldsymbol{X}^{(1)}-\boldsymbol{X}^{(1)}W_0^{(1)}\right)_1&=\boldsymbol{X}_1^{(1)}\\&=\boldsymbol{\epsilon}_1^{(1)}\\\left(\boldsymbol{X}^{(1)}-\boldsymbol{X}^{(1)}W_0^{(1)}\right)_2&=\boldsymbol{X}_2^{(1)}-w\boldsymbol{X}_1^{(1)}\\&=\boldsymbol{X}_2^{(1)}-w\boldsymbol{X}_1^{(1)}\\&=w\boldsymbol{\epsilon}_1^{(1)}+\boldsymbol{\epsilon}_2^{(1)}-w\boldsymbol{\epsilon}_1^{(1)}\\&=\boldsymbol{\epsilon}_2^{(1)}\end{aligned}$$

which gives us the expected component-wise losses

$$\begin{aligned}\frac{1}{n_1}\frac{\alpha^2}{\sigma_1^2}\mathbb{E}\left|\left|\left(\boldsymbol{X}^{(1)}-\boldsymbol{X}^{(1)}W_0^{(1)}\right)_1\right|\right|_F^2&=\frac{\alpha^2}{\sigma_1^2}\frac{\sigma_1^2}{\alpha^2}\\&=1\\\frac{1}{n_1}\frac{\alpha^2}{\sigma_2^2}\mathbb{E}\left|\left|\left(\boldsymbol{X}^{(1)}-\boldsymbol{X}^{(1)}W_0^{(1)}\right)_2\right|\right|_F^2&=\frac{\alpha^2}{\sigma_2^2}\sigma_2^2\\&=\alpha^2\end{aligned}$$

and thus

$$\mathbb{E}\mathcal{L}_{\Omega_\alpha}\left(W_0^{(1)},\boldsymbol{X}^{(1)}\right)=1+\alpha^2.$$

Note that under intervention on X_1, $W_\delta^{(1)} = \begin{pmatrix} 0 & 0 \\ 0 & 0 \end{pmatrix}$. Component-wise, we then have

$$\begin{aligned}
\left(\boldsymbol{X}^{(1)} - \boldsymbol{X}^{(1)} W_\delta^{(1)}\right)_1 &= \boldsymbol{X}_1^{(1)} \\
&= \boldsymbol{\epsilon}_1^{(1)} \\
\left(\boldsymbol{X}^{(1)} - \boldsymbol{X}^{(1)} W_\delta^{(1)}\right)_2 &= \boldsymbol{X}_2^{(1)} \\
&= w\boldsymbol{\epsilon}_1^{(1)} + \boldsymbol{\epsilon}_2^{(1)}
\end{aligned}$$

and the expected component-wise loss

$$\begin{aligned}
\frac{1}{n_1}\frac{\alpha^2}{\sigma_1^2}\mathbb{E}\left|\left|\left(\boldsymbol{X}^{(1)} - \boldsymbol{X}^{(1)} W_\delta^{(1)}\right)_1\right|\right|_F^2 &= \frac{\alpha^2}{\sigma_1^2}\frac{\sigma_1^2}{\alpha^2} \\
&= 1 \\
\frac{1}{n_1}\frac{\alpha^2}{\sigma_2^2}\mathbb{E}\left|\left|\left(\boldsymbol{X}^{(1)} - \boldsymbol{X}^{(1)} W_\delta^{(1)}\right)_2\right|\right|_F^2 &= \frac{\alpha^2}{\sigma_2^2}\left(w^2\frac{\sigma_1^2}{\alpha^2} + \sigma_2^2\right) \\
&= \alpha^2\left(w^2\frac{\sigma_1^2}{\sigma_2^2} + 1\right).
\end{aligned}$$

Thus,

$$\mathbb{E}\mathcal{L}_{\Omega_\alpha}\left(W_\delta^{(1)}, \boldsymbol{X}^{(1)}\right) = 1 + \alpha^2 + \alpha^2 w^2 \frac{\sigma_1^2}{\sigma_2^2} > \mathbb{E}\mathcal{L}_{\Omega_\alpha}\left(W_0^{(1)}, \boldsymbol{X}^{(1)}\right),$$

which proves the result.

S1.3.3. Intervention on node 2

Under intervention on X_2, $W_0^{(2)} = \begin{pmatrix} 0 & 0 \\ 0 & 0 \end{pmatrix}$, with corresponding SEM

$$\begin{aligned}
X_1^{(2)} &= \epsilon_1^{(2)} \\
X_2^{(2)} &= \epsilon_2^{(2)}.
\end{aligned}$$

Component-wise, we obtain the terms

$$\begin{aligned}
\left(\boldsymbol{X}^{(2)} - \boldsymbol{X}^{(2)} W_0^{(2)}\right)_1 &= \boldsymbol{X}_1^{(2)} \\
&= \boldsymbol{\epsilon}_1^{(2)} \\
\left(\boldsymbol{X}^{(2)} - \boldsymbol{X}^{(2)} W_0^{(2)}\right)_2 &= \boldsymbol{X}_2^{(2)} \\
&= \boldsymbol{\epsilon}_2^{(2)}
\end{aligned}$$

Note that $\mathrm{Var}\left(\epsilon_2^{(2)}\right) = \frac{\sigma_2}{\alpha^2}$ in accordance with Assumption 3. Then the expected loss component-wise is

$$\begin{aligned}
\frac{1}{n_2}\frac{\alpha^2}{\sigma_1^2}\mathbb{E}\left|\left|\left(\boldsymbol{X}^{(2)} - \boldsymbol{X}^{(2)} W_0^{(2)}\right)_1\right|\right|_F^2 &= \frac{\alpha^2}{\sigma_1^2}\sigma_1^2 \\
&= \alpha^2 \\
\frac{1}{n_2}\frac{\alpha^2}{\sigma_2^2}\mathbb{E}\left|\left|\left(\boldsymbol{X}^{(2)} - \boldsymbol{X}^{(2)} W_0^{(2)}\right)_2\right|\right|_F^2 &= \frac{\alpha^2}{\sigma_2^2}\frac{\sigma_2^2}{\alpha^2} \\
&= 1,
\end{aligned}$$

giving

$$\mathbb{E}\mathcal{L}_{\Omega_\alpha}\left(W_0^{(2)}, \boldsymbol{X}^{(2)}\right) = 1 + \alpha^2.$$

Under $W_\delta^{(2)} = \begin{pmatrix} 0 & 0 \\ \delta & 0 \end{pmatrix}$, we obtain the terms component-wise

$$\begin{aligned}\left(\boldsymbol{X}^{(2)} - \boldsymbol{X}^{(2)} W_\delta^{(2)}\right)_1 &= \boldsymbol{X}_1^{(2)} - \delta \boldsymbol{X}_2^{(2)} \\ &= \boldsymbol{\epsilon}_1^{(2)} - \delta \boldsymbol{\epsilon}_2^{(2)} \\ \left(\boldsymbol{X}^{(2)} - \boldsymbol{X}^{(2)} W_\delta^{(2)}\right)_2 &= \boldsymbol{X}_2^{(2)} \\ &= \boldsymbol{\epsilon}_2^{(2)}\end{aligned}$$

which in expectation gives the component-wise losses

$$\begin{aligned}\frac{1}{n_2}\frac{\alpha^2}{\sigma_1^2}\mathbb{E}\left|\left|\left(\boldsymbol{X}^{(2)} - \boldsymbol{X}^{(2)} W_\delta^{(2)}\right)_1\right|\right|_F^2 &= \frac{\alpha^2}{\sigma_1^2}\left(\sigma_1^2 + \delta^2 \frac{\sigma_2^2}{\alpha^2}\right) \\ &= \alpha^2 + \delta^2 \frac{\sigma_2^2}{\sigma_1^2} \\ \frac{1}{n_2}\frac{\alpha^2}{\sigma_2^2}\mathbb{E}\left|\left|\left(\boldsymbol{X}^{(2)} - \boldsymbol{X}^{(2)} W_\delta^{(2)}\right)_2\right|\right|_F^2 &= \frac{\alpha^2}{\sigma_2^2}\frac{\sigma_2^2}{\alpha^2},\end{aligned}$$

which gives

$$\mathbb{E}\mathcal{L}_{\Omega_\alpha}\left(W_\delta^{(2)}, \boldsymbol{X}^{(2)}\right) = 1 + \alpha^2 + \delta^2 \frac{\sigma_2^2}{\sigma_1^2}$$

which is trivially greater than $\mathbb{E}\mathcal{L}_{\Omega_\alpha}\left(W_\delta^{(2)}, \boldsymbol{X}^{(2)}\right)$ for all $\alpha, \delta, \sigma_1^2, \sigma_2^2$.

S1.4. Full two node simulation details

In Section 3.1.1, we simulate from the system of SEMs

$$\begin{aligned}X^{(0)} &= X^{(0)} W + \epsilon^{(0)} \\ X^{(1)} &= X^{(1)} W^{(1)} + \epsilon^{(1)} \\ X^{(2)} &= X^{(2)} W^{(2)} + \epsilon^{(2)},\end{aligned}$$

such that in the observational system

$$\begin{aligned}X_1^{(0)} &= \epsilon_1^{(1)} & \epsilon_1^{(0)} &\sim \mathcal{N}(0, \gamma) \\ X_2^{(0)} &= w X_1^{(0)} + \epsilon_2^{(0)} & \epsilon_2^{(0)} &\sim \mathcal{N}(0, 1),\end{aligned} \tag{34}$$

under intervention on node 1 we have

$$\begin{aligned}X_1^{(1)} &= \epsilon_1^{(1)} & \epsilon_1^{(1)} &\sim \mathcal{N}\left(0, \frac{\gamma}{\alpha^2}\right) \\ X_2^{(1)} &= w X_1^{(1)} + \epsilon_2^{(1)} & \epsilon_2^{(1)} &\sim \mathcal{N}(0, 1),\end{aligned} \tag{35}$$

and under intervention on node 2

$$\begin{aligned}X_1^{(2)} &= \epsilon_1^{(2)} & \epsilon_1^{(2)} &\sim \mathcal{N}(0, \gamma) \\ X_2^{(2)} &= \epsilon_2^{(2)} & \epsilon_2^{(2)} &\sim \mathcal{N}\left(0, \frac{1}{\alpha^2}\right).\end{aligned} \tag{36}$$

For observational data, we simulate only from the SEM given in Eq. 34, with a sample size $n = 3000$. For interventional data, we draw a sample size of $n_k = 1000$ from each intervention k. This gives a total sample size of $n = (p+1) \times 3000$ for interventional data, which matches the sample size of observational data. We set $\alpha = 4$. In Section 3.1.1, we presented empirical results on two node simulations for $(w, \gamma) \in \{0.3, 0.5, 0.7, 0.8, 1.0\} \times \{1, 2, 100\}$. Figure S1 shows the ℓ_1 distance between the ground truth DAG W_0 and the inferred DAG for each method, for all $(w, \gamma) \in \{0.1, 0.2, \ldots, 1.5\} \times \{1, 2, 4, 10, 100\}$. For each parameter combination of (w, γ), we draw 25 instances of simulated data for both the interventional and strictly observational case, at the described sample size.

For `dotears`, sortnregress, and NO TEARS, we set the regularization parameter λ to 0, to isolate the performance of the loss function. In GOLEM-EV and GOLEM-NV we set the ℓ_1 regularization parameter λ_1 to 0, but let $\lambda_2 = 5$ to enforce DAG-ness, as recommended by the authors.

See Figure S1 for the full set of simulated w, γ.

S1.5. Full three node simulations

Simulations in the two-node DAG showed that NO TEARS and GOLEM-NV are deterministic functions of the true structure W and the true exogenous variance structure Ω_0 (Sections 3.1.1, Supplementary Material S1.4). Simulations in more complex three-node topologies verify determinism of NO TEARS and GOLEM-NV in W and Ω_0, but show they are distinct deterministic functions.

We simulate observational and interventional data under the three node chain $X_1 \overset{w}{\to} X_2 \overset{w}{\to} X_3$, the three node collider $X_1 \overset{w}{\to} X_3 \overset{w}{\leftarrow} X_2$, and the three node fork $X_2 \overset{w}{\leftarrow} X_1 \overset{w}{\to} X_3$. In each topology, we let $\Omega_0 := \begin{pmatrix} \gamma & 0 & 0 \\ 0 & 1 & 0 \\ 0 & 0 & 1 \end{pmatrix}$, so that γ is the exogenous variance of a source node. For simplicity, we constrain the edge weights to be equal, and simulate data under Gaussian exogenous variance for $(w, \gamma) \in \{0.1, \ldots, 1.5\} \times \{1, 2, 4, 10, 100\}$. For interventional data with interventions $i \in \{0, 1, 2, 3\}$, we draw $n_i = 1000$ observations in all simulations, giving us a total sample size of $n = 4000$ that is matched in observational data. As in section 3.1.1, we set $\alpha = 4$, and remove ℓ_1 regularization where appropriate. We benchmark `dotears`, NO TEARS, sortnregress, GOLEM-EV, GOLEM-NV, DirectLingam, GIES, IGSP, UT-IGSP, and DCDI-G.[10,12,15,13,21,22,23,14,24]

We evaluate each method by the SHD between the ground truth DAG W and the method inferred DAG. In the two node case, the least squares loss performed identically to the likelihood loss of GOLEM-NV; here, their performances diverge, but remain essentially deterministic functions in w and γ.

S1.5.1. Chain

In the chain, we have the true structure $W_0 = \begin{pmatrix} 0 & w & 0 \\ 0 & 0 & w \\ 0 & 0 & 0 \end{pmatrix}$. We simulate under the system of SEMs

$$\begin{aligned} X^{(0)} &= X^{(0)} W_0 + \epsilon^{(0)} \\ X^{(1)} &= X^{(1)} W_0^{(1)} + \epsilon^{(1)} \\ X^{(2)} &= X^{(2)} W_0^{(2)} + \epsilon^{(2)}, \\ X^{(3)} &= X^{(3)} W_0^{(3)} + \epsilon^{(3)}, \end{aligned}$$

such that in the observational system

$$\begin{aligned} X_1^{(0)} &= \epsilon_1^{(1)} & \epsilon_1^{(0)} &\sim \mathcal{N}(0, \gamma) \\ X_2^{(0)} &= w X_1^{(0)} + \epsilon_2^{(0)} & \epsilon_2^{(0)} &\sim \mathcal{N}(0, 1), \\ X_3^{(0)} &= w X_2^{(0)} + \epsilon_3^{(0)} & \epsilon_3^{(0)} &\sim \mathcal{N}(0, 1), \end{aligned}$$

under intervention on node 1 we have

$$
\begin{aligned}
X_1^{(1)} &= \epsilon_1^{(1)} & \epsilon_1^{(1)} &\sim \mathcal{N}\left(0, \frac{\gamma}{\alpha^2}\right) \\
X_2^{(1)} &= wX_1^{(1)} + \epsilon_2^{(1)} & \epsilon_2^{(1)} &\sim \mathcal{N}\left(0, 1\right), \\
X_3^{(1)} &= wX_2^{(1)} + \epsilon_3^{(1)} & \epsilon_3^{(1)} &\sim \mathcal{N}\left(0, 1\right),
\end{aligned}
$$

under intervention on node 2

$$
\begin{aligned}
X_1^{(2)} &= \epsilon_1^{(2)} & \epsilon_1^{(2)} &\sim \mathcal{N}\left(0, \gamma\right) \\
X_2^{(2)} &= \epsilon_2^{(2)} & \epsilon_2^{(2)} &\sim \mathcal{N}\left(0, \frac{1}{\alpha^2}\right), \\
X_3^{(2)} &= wX_2^{(2)} + \epsilon_3^{(2)} & \epsilon_3^{(2)} &\sim \mathcal{N}\left(0, 1\right),
\end{aligned}
$$

and under intervention on node 3

$$
\begin{aligned}
X_1^{(3)} &= \epsilon_1^{(3)} & \epsilon_1^{(3)} &\sim \mathcal{N}\left(0, \gamma\right) \\
X_2^{(3)} &= wX_1^{(3)} + \epsilon_2^{(3)} & \epsilon_2^{(3)} &\sim \mathcal{N}\left(0, 1\right), \\
X_3^{(1)} &= \epsilon_3^{(3)} & \epsilon_3^{(3)} &\sim \mathcal{N}\left(0, \frac{1}{\alpha^2}\right).
\end{aligned}
$$

Figure S2 shows the full results.

S1.5.2. Collider

In the collider, we have the true structure $W_0 = \begin{pmatrix} 0 & 0 & w \\ 0 & 0 & w \\ 0 & 0 & 0 \end{pmatrix}$. We simulate under the system of SEMs

$$
\begin{aligned}
X^{(0)} &= X^{(0)}W_0 + \epsilon^{(0)} \\
X^{(1)} &= X^{(1)}W_0^{(1)} + \epsilon^{(1)} \\
X^{(2)} &= X^{(2)}W_0^{(2)} + \epsilon^{(2)}, \\
X^{(3)} &= X^{(3)}W_0^{(3)} + \epsilon^{(3)},
\end{aligned}
$$

such that in the observational system

$$
\begin{aligned}
X_1^{(0)} &= \epsilon_1^{(1)} & \epsilon_1^{(0)} &\sim \mathcal{N}\left(0, \gamma\right) \\
X_2^{(0)} &= \epsilon_2^{(0)} & \epsilon_2^{(0)} &\sim \mathcal{N}\left(0, 1\right), \\
X_3^{(0)} &= wX_1^{(0)} + wX_2^{(0)} + \epsilon_3^{(0)} & \epsilon_3^{(0)} &\sim \mathcal{N}\left(0, 1\right),
\end{aligned}
$$

under intervention on node 1 we have

$$
\begin{aligned}
X_1^{(1)} &= \epsilon_1^{(1)} & \epsilon_1^{(1)} &\sim \mathcal{N}\left(0, \frac{\gamma}{\alpha^2}\right) \\
X_2^{(1)} &= \epsilon_2^{(1)} & \epsilon_2^{(1)} &\sim \mathcal{N}\left(0, 1\right), \\
X_3^{(1)} &= wX_1^{(1)} + wX_2^{(1)} + \epsilon_3^{(1)} & \epsilon_3^{(1)} &\sim \mathcal{N}\left(0, 1\right),
\end{aligned}
$$

under intervention on node 2

$$\begin{aligned} X_1^{(2)} &= \epsilon_1^{(2)} & \epsilon_1^{(2)} &\sim \mathcal{N}(0, \gamma) \\ X_2^{(2)} &= \epsilon_2^{(2)} & \epsilon_2^{(2)} &\sim \mathcal{N}\left(0, \frac{1}{\alpha^2}\right), \\ X_3^{(2)} &= wX_1^{(2)} + wX_2^{(2)} + \epsilon_3^{(2)} & \epsilon_3^{(2)} &\sim \mathcal{N}(0, 1), \end{aligned}$$

and under intervention on node 3

$$\begin{aligned} X_1^{(3)} &= \epsilon_1^{(3)} & \epsilon_1^{(3)} &\sim \mathcal{N}(0, \gamma) \\ X_2^{(3)} &= \epsilon_2^{(3)} & \epsilon_2^{(3)} &\sim \mathcal{N}(0, 1), \\ X_3^{(1)} &= \epsilon_3^{(3)} & \epsilon_3^{(3)} &\sim \mathcal{N}\left(0, \frac{1}{\alpha^2}\right). \end{aligned}$$

S1.5.3. Fork

In the collider, we have the true structure $W_0 = \begin{pmatrix} 0 & w & w \\ 0 & 0 & 0 \\ 0 & 0 & 0 \end{pmatrix}$. We simulate under the system of SEMs

$$\begin{aligned} X^{(0)} &= X^{(0)} W_0 + \epsilon^{(0)} \\ X^{(1)} &= X^{(1)} W_0^{(1)} + \epsilon^{(1)} \\ X^{(2)} &= X^{(2)} W_0^{(2)} + \epsilon^{(2)}, \\ X^{(3)} &= X^{(3)} W_0^{(3)} + \epsilon^{(3)}, \end{aligned}$$

such that in the observational system

$$\begin{aligned} X_1^{(0)} &= \epsilon_1^{(1)} & \epsilon_1^{(0)} &\sim \mathcal{N}(0, \gamma) \\ X_2^{(0)} &= wX_1^{(0)} + \epsilon_2^{(0)} & \epsilon_2^{(0)} &\sim \mathcal{N}(0, 1), \\ X_3^{(0)} &= wX_1^{(0)} + \epsilon_3^{(0)} & \epsilon_3^{(0)} &\sim \mathcal{N}(0, 1), \end{aligned}$$

under intervention on node 1 we have

$$\begin{aligned} X_1^{(1)} &= \epsilon_1^{(1)} & \epsilon_1^{(1)} &\sim \mathcal{N}\left(0, \frac{\gamma}{\alpha^2}\right) \\ X_2^{(1)} &= wX_1^{(0)} + \epsilon_2^{(1)} & \epsilon_2^{(1)} &\sim \mathcal{N}(0, 1), \\ X_3^{(1)} &= wX_1^{(1)} + \epsilon_3^{(1)} & \epsilon_3^{(1)} &\sim \mathcal{N}(0, 1), \end{aligned}$$

under intervention on node 2

$$\begin{aligned} X_1^{(2)} &= \epsilon_1^{(2)} & \epsilon_1^{(2)} &\sim \mathcal{N}(0, \gamma) \\ X_2^{(2)} &= \epsilon_2^{(2)} & \epsilon_2^{(2)} &\sim \mathcal{N}\left(0, \frac{1}{\alpha^2}\right), \\ X_3^{(2)} &= wX_1^{(2)} + \epsilon_3^{(2)} & \epsilon_3^{(2)} &\sim \mathcal{N}(0, 1), \end{aligned}$$

and under intervention on node 3

$$\begin{aligned} X_1^{(3)} &= \epsilon_1^{(3)} & \epsilon_1^{(3)} &\sim \mathcal{N}(0,\gamma) \\ X_2^{(3)} &= wX_1^{(3)} + \epsilon_2^{(3)} & \epsilon_2^{(3)} &\sim \mathcal{N}(0,1), \\ X_3^{(1)} &= \epsilon_3^{(3)} & \epsilon_3^{(3)} &\sim \mathcal{N}\left(0, \frac{1}{\alpha^2}\right). \end{aligned}$$

S1.6. Incorporation of interventional loss

We note that inferring W from strictly observational data, given $\hat{\Omega}_0$ from interventional data, has convenient theoretical properties but ignores the majority of our data. Figure S10 compares the performance, in Structural Hamming Distance, of `dotears` using both observational and interventional data $\left(\mathcal{L}_{\hat{\Omega}_0}\left(W^{(k)}, \boldsymbol{X}^{(k)}\right) \text{ for } k = 0 \ldots p\right)$ with `dotears` using only observational data $\left(\mathcal{L}_{\hat{\Omega}_0}\left(W^{(k)}, \boldsymbol{X}^{(k)}\right) \text{ for } k = 0\right)$. We use the large random graph simulations described above, aggregated across all scenarios in Table S1. Note that $\hat{\Omega}_0$ is estimated identically for both.

As expected, restricting `dotears` to inference only using the observational data (Figure S10, right) drastically decreases performance when compared to inference using both observational and interventional data (Figure S10, left). This motivates including $k = 1 \ldots p$ into the loss and proving consistency for all k.

S1.7. Sensitivity Analysis

S1.7.1. Sensitivity under linear SEM

We performed sensitivity analyses under a linear SEM on Erdős-Rényi graphs ($p = 20$) with two edge density parameterizations crossed with two edge weight parameterizations (we note that the specific density and edge weight parameterizations differ from Section 3.2). We report results averaged over 10 replicates with a sample size of $n_k = 100$. We report SHD in the main text. For precision/recall for edge recovery, parameterization-specific results, details on data generation, and modelling specifics, see Supplementary Material S1.8.

We assessed the sensitivity of our results to violation of Assumption 1 by allowing for π percent of the observational parental variance into the marginal variance of the target . Here, $\pi = 0$ is a hard intervention. The accuracy of `dotears` decreases as π increases, or equivalently as the interventional signal decreases (Figure S12a). This effect is dependent on the magnitude of the edge weights and the density of the true DAG: in "High Density" and "Strong Effects" scenarios, the decrease in accuracy is noticeable due to higher incoming parental variance (Supplementary Figure S13). Despite this decrease in accuracy, `dotears` remains the second best performing method across parameterizations, even when excluding the $\pi = 0$ scenario.

To assess sensitivity to violation of Assumption 3, we randomly perturb each α per node to allow non-uniform interventional effects on error variance across targets. `dotears` is robust to violations of Assumption 3 in these simulations (Figure S12b).

Finally, our model assumes that the distribution of the target $X_k^{(k)}$ depends on that of $\epsilon_k^{(0)}$. In Figure S12c, we explore interventions with a fixed distribution, $X_k^{(k)} \overset{iid}{\sim} \mathcal{N}(2,1)$ following the model used in Brouillard *et al.*[14] and similar to that considered by Hauser *et al.*[10] In our model, this violates Assumption 3.

In Figure S12c, `dotears` is outperformed on average only by GIES in SHD, and outperforms DCDI under their simulation model. Supplementary Figure S16 shows that simulations with weak effects, where $0.3 \leq |w| \leq 0.5$ drive this performance dip; in simulations with strong effects, where $0.8 \leq |w| \leq 1.0$, `dotears` again vastly outperforms other methods, including GIES. Sufficient signal-to-noise ratio can thus overcome severe misspecification of Ω_0 under hard interventions.

S1.7.2. Sensitivity under nonlinear SEM

To assess sensitivity to a nonlinear SEM, we generate nonlinear data under models that were used to evaluate DCDI.[14] We simulated 10 Erdős-Rényi DAGs with $p = 10$ and $n = 10000$ total samples. For the non-linear models, we considered, in turn, a neural network additive noise model (ANM)[32] and a neural network with non-additive noise model (NN).[33] We test both hard interventions and "imperfect" interventions.

Imperfect interventions add a random vector drawn from $\mathcal{N}(0, 1)$ to the last layer of the neural network. See[14] and Supplementary Material S1.9 for details. Figure S11 shows results in SHD.

With hard interventions, `dotears` maintains performance in DAG estimation across increasing levels of non-linearity, and on average outperforms all methods. `dotears` performs worse in the "imperfect" intervention setting. In particular, `dotears` has average performance in "Low Density" scenarios, but performs much worse in "High Density" simulations. This suggests that the imperfect intervention model, rather than non-linearity, drives the relatively worse performance of `dotears`. As in Figure S12a, dense DAGs exacerbate difficulties with imperfect interventions. However, on average `dotears` outperforms the neural network DCDI even under "imperfect" interventions.

S1.8. Sensitivity under linear SEM

For evaluation of `dotears` sensitivity to violations of the modeling assumptions, data is generated for DAGs with $p = 20$ nodes. Structures are drawn from both Erdős-Rényi (ER) and Scale-Free (SF) DAGs.[25,26] We simulate under two parameterizations {Low Density, High Density} of the edge densities (r, z). In the "Low Density" parameterization, give $(r, z) = (0.2, 2)$. To evaluate performance on higher density topologies, we also give "High Density" parameterizations, where $(r, z) = (0.5, 6)$.

Given an edge density scenario, we simulate under two parameterizations of the edge weights. In the "Strong Effects" parameterization, $w \sim \text{Unif}([-1.0, -0.8] \cup [0.8, 1.0])$. We also give the "Weak Effects" parameterization, which edge weights are drawn from $w \sim \text{Unif}([-0.3, -0.5] \cup [0.3, 0.5])$. Table S1 summarizes all four possible simulation parameterizations.

For each node i, we draw $\sigma_i \sim \text{Unif}([0.5, 2.0])$, and draw n_0 observations from $\boldsymbol{\epsilon}_i^{(0)} \sim \mathcal{N}\left(0, \sigma_i^2\right)$. For each DAG we generate an instance of observational data, where $n_0 = (p + 1) * n_k = 2100$, and an instance of interventional data, where $n_k = 100$ for all $k = 0 \ldots p$ to match sample size. We set the distribution of $\epsilon_i^{(k)}$ according to Assumptions 3 and 2 for $\alpha = 4$. Cross-validation was performed as in STAR Methods.

S1.8.1. Hard Interventions

Assumption 1 assumes that interventions remove all edges incoming to the target, and therefore that the marginal variance of the target has no incoming parental variance. We examine the model where

$$X_k^{(k)} = \sqrt{\pi} \sum_{i=0}^{p} w_{ij} X_i^{(k)} + \epsilon_k^{(k)},$$

where interventions downweight incoming parental edges by $\sqrt{\pi}$. Accordingly, this means that

$$\text{Var}\left(X_k^{(k)}\right) = \pi \sum_{i=0}^{p} w_{ij}^2 \text{Var}\left(X_i^{(k)}\right) + \frac{\sigma_k^2}{\alpha^2},$$

where for $\pi = 0$ we recover hard interventions. We call these interventions either soft or imperfect interventions, and examine method performance when $\pi = \{0, 0.1, 0.25, 0.5\}$. Note that other than the imperfect intervention, the model and generating processes are the same as in STAR Methods.

For each $\pi \in \{0, 0.1, 0.25, 0.5\}$ and each parameterization in Table S3, ten Erdős-Rényi DAGs and ten Scale-Free DAGs are drawn, with sample size matched as described above. Results in Structural Hamming Distance are shown in Supplementary Figure S13 per parameterization, and are shown averaged across parameterizations in Figure S12a. Results in binary precision and recall are given in Supplementary Figure S14 and Supplementary Figure S15, respectively.

S1.8.2. α perturbation

Assumption 3 assumes that for each node under intervention, the error variance is reduced by a shared factor α across interventional targets, i.e.

$$\text{Var}\left(\epsilon_k^{(k)}\right) = \frac{\sigma_k^2}{\alpha^2}$$

for all k.

To test the sensitivity of `dotears` to this assumption, we draw data for which

$$\mathrm{Var}\left(\epsilon_k^{(k)}\right) = \frac{\sigma_k^2}{c_k^2\alpha^2}, \quad c_k \sim \mathrm{Unif}([0.8, 1.2]).$$

c_k is target-specific, and thus under this model Ω_0 will be misspecified.

Ten Erdős-Rényi DAGs and ten Scale-Free DAGs are drawn, with sample size matched as described above. Results in Structural Hamming Distance, averaged across parameterizations, are shown in Figure S12b. Results for each parameterization in Structural Hamming Distance are shown in Supplementary Figure S16 (top row). Results in binary edge precision are shown in Supplementary Figure S17. Results in binary edge recall are shown in Supplementary Figure S18.

S1.8.3. Fixed Interventions

Our model assumes that the distribution of node k under intervention on k is dependent on the distribution of $\epsilon_k^{(0)}$. We also examine an interventional model in which intervention on a node k sets the distribution with a fixed mean shift and error variance, i.e.

$$X_k^{(k)} \sim \mathcal{N}(2, 1),$$

or equivalently $\epsilon_k^{(k)}$. This model is used in linear simulations in Brouillard *et al.*[14] and is similar to that of.[10]

S1.9. Sensitivity to nonlinear SEM

To evaluate the performance of `dotears` on nonlinear data, we generate data from the generative models in.[14] Specifically, structures are Erdős-Rényi random DAGs with $p = 10$ with single-node interventions on every node. The total sample size is $n = 10000$ for all draws and scenarios, evenly split across the interventional scenarios (10 interventions and 1 observational) for $n_k \approx 909$. Only 5 samples are drawn from each scenario due to runtime limitations.

Data is generated either from the additive noise model (ANM) or the non-linear with non-additive noise model (NN). For a node j, let $N_j \sim \mathcal{N}(0, \sigma_j^2)$, where $\sigma_j^2 \sim \mathrm{Unif}[1, 2]$. In the additive noise model, for a node j we have

$$X_j := f_j\left(\mathrm{Pa}(j)\right) + 0.4 \cdot N_j, \tag{37}$$

where "f_j are fully connected neural networks with one hidden layer of 10 units and *leaky ReLU* with a negative slope of 0.25 as nonlinearities".[14] Imperfect interventions add a random vector of $\mathcal{N}(0, 1)$ to the last layer; for details, see.[14]

In the nonlinear with non-additive noise model, for a node j we have

$$X_j := f_j\left(\mathrm{Pa}(X_j), N_j\right), \tag{38}$$

where f_j are "fully connected neural networks with one hidden layer of 20 units and *tanh* as nonlinearities".[14] Imperfect interventions are obtained in the same fashion as the additive noise model; for details, see.[14]

We give two parameterizations for each setting: "Low Density", in which the expected number of edges is 10, and "High Density", in which the expected number of edges is 40. Results in SHD are given in Figure S11. Results in precision and recall are given in Figure S19 and Figure S20, respectively.

References

[31] Yuhao Wang, Chandler Squires, Anastasiya Belyaeva, and Caroline Uhler. Direct estimation of differences in causal graphs. *Advances in neural information processing systems*, 31, 2018.

[32] Peter Bühlmann, Jonas Peters, and Jan Ernest. Cam: Causal additive models, high-dimensional order search and penalized regression. 2014.

[33] David Maxwell Chickering. Optimal structure identification with greedy search. *Journal of machine learning research*, 3(Nov):507–554, 2002
